# “*All the fun stuff, the teachers say, ‘that’s dangerous!*’” Hearing from children on safety and risk in active play in schools: a systematic review

**DOI:** 10.1186/s12966-022-01305-0

**Published:** 2022-06-25

**Authors:** Alethea Jerebine, Katie Fitton-Davies, Natalie Lander, Emma L. J. Eyre, Michael J. Duncan, Lisa M. Barnett

**Affiliations:** 1 School of Health and Social Development, Faculty of Health, Deakin University, VIC Geelong, Australia; 2 Centre for Sport, Exercise and Life Sciences, Coventry University, Coventry, UK; 3 Research Institute for Sport and Exercise Sciences, Liverpool John Moores University, Liverpool, UK; 4 Institute for Physical Activity and Nutrition, School of Exercise and Nutrition Sciences, Deakin University, Geelong, VIC Australia; 5 Institute for Physical Activity and Nutrition, Deakin University, Geelong, VIC Australia

**Keywords:** Risky play, Physical activity, Recess, Affordance theory, Social-ecological model, Physical literacy, Qualitative, Risk tolerance

## Abstract

**Background:**

Active play is vital for healthy child development, and schools are a valuable setting to promote this behaviour. Understanding the determinants of children’s physical activity behaviour during recess, particularly the role of risk-taking and the influence safety concerns have on active play, is required. This systematic review aimed to 1) synthesise qualitative research with children that explored their perceptions of safety and risk in active play during recess in elementary and/or middle school, and 2) develop a model from the findings to guide efforts in schools to optimise children’s active play opportunities during recess.

**Methods:**

Six online databases were systematically searched for articles published between January 2000 and March 2021. Following PRISMA guidelines, records were screened against eligibility criteria using Covidence software, and data extraction and synthesis was conducted using customised forms in Excel and NVivo software. Framework synthesis methodology was employed, conceptually guided by Bronfenbrenner’s socio-ecological model and Gibson’s affordance theory.

**Results:**

Of 9664 records, 31 studies met inclusion criteria, representing 1408 children across 140 schools from 11 countries. An emergent conceptual framework was developed encompassing 23 risk and safety themes and 10 risky play types that children desired in schools. Individual characteristics (age, gender, physical literacy) influenced children’s engagement with risk and how they kept themselves safe. Across outer SEM levels, factors interacted to constrain or afford children’s active play. Socio-cultural factors (supervision practices, rules, equipment restrictions) constrained active play, which children perceived were driven by adults’ concern with physical safety. These factors contributed to a cycle of risk-averse decision making and diminished play affordances, which could inadvertently exacerbate safety issues. A model for risk tolerance in children’s active play has been proposed.

**Conclusions:**

The findings show a disparity between the active play children want in schools and what they are able to do. Future work should balance the concerns of adults against the active play children want, involve children in decisions about playground policy, and foster a risk-tolerant culture in schools.

**Supplementary Information:**

The online version contains supplementary material available at 10.1186/s12966-022-01305-0.

## Introduction

Regular physical activity (PA) is essential for healthy child development, including musculoskeletal development, cardiovascular health, and mental wellbeing [1–3], with growing evidence for cognitive and academic benefits [4–6]. Despite these benefits, children’s PA levels remain persistently low and may even be decreasing in some nations [7–10]. Play is a universal expression of childhood and, like PA, has a fundamental role in the psychological, social, physical, and cognitive development of children [11–14]. The United Nations Convention on the Rights of the Child (CRC) includes the right to play and equitable access to play [15]. Play is an important domain of children’s PA [16], and is commonly described as ‘active play’ [17, 18], although considerable variability exists [19]. The definition proposed by Truelove and colleagues is adopted in this review: “*active play is a form of gross motor or total body movement in which children exert energy in a freely chosen, fun, and unstructured manner*” ([17], p.164).

Schools are a valuable setting to promote children’s health and wellbeing, including active play, as most children spend significant time in school, usually with dedicated periods set aside for free play outdoors [20, 21]. For children without access to outdoor play spaces (i.e., backyards, local parks), or with busy after-school schedules, school may represent their only opportunity for regular active play outdoors [16, 22–24]. Despite most countries being signatories to the CRC [25], many fail to protect children’s rights to play at school through legislation. For example, in the US, only eight states require schools to provide breaks between lessons (i.e. recess) [26], while in the UK [27], Canada [28], and Australia [29] there is no nationally mandated requirement. Arguably, one impact of this is the negative trend in time allocated to recess in recent decades [26–28]. There is also concern globally that the Covid-19 pandemic may have reduced play opportunities further [30].

Despite school being the most researched of children’s PA settings, efforts to improve children’s school-based PA have experienced variable success [31]. Studies seeking to understand the factors that influence children’s PA during play can provide important insights into determinants of behaviour [32, 33], however, much of the research that has investigated children’s school recess behaviour has been oriented towards increasing children’s *PA* [34, 35], with less attention to *play*, per se [36]. This narrowing of focus may have inadvertently excluded important drivers of children’s PA behaviour during play, particularly for less active or ‘sporty’ children [37]. For instance, there is a growing body of literature on the role of risk-taking and challenge in children’s PA and play [38–40], and the impact safety concerns and risk-averse decision making in the school system have on the social and physical play environment children experience [41, 42].

In recent decades, concerns for children’s safety have increased in line with increasing societal aversion to risk [43–45]. Social norms oriented towards protecting children from all possible harms have emerged [41, 46, 47], leading to declining opportunities for play outdoors [36, 48, 49], and increasing monitoring and surveillance [12]. Moreover, in many western countries, concerns about risk minimisation have resulted in safety legislation for children’s play environments [44, 50, 51], with contemporary play landscapes engineered to remove all risk and challenge, leaving a “KFC” playground, containing a **K**it of prefabricated play equipment, a **F**ence, and a **C**arpet of rubber safety surfacing [37, 52]. Alongside this cultural shift has been an increasing interest in the concept of ‘risky play’, which aims to articulate the inclination children have for risk-taking and challenge in play, and its significance for healthy child development [39, 40, 42]. Sandseter’s ([53], p.22) widely used definition describes risky play as “*thrilling and exciting forms of physical play that involve uncertainty and a risk of physical injury*”. Risky play primarily takes place outdoors, often in the form of challenging and vigorous physical activities, providing children with opportunities to push themselves, test physical limits, and experience the satisfaction of mastering new skills [39, 54, 55]. Evidence for children’s inclination for risky play, as a necessary and natural part of active play, is increasing, including its positive influence on PA [38, 56].

Research documenting the influence of disproportionate safety concerns on children’s PA and play outdoors is also growing. Qualitative systematic reviews have identified risk-averse behaviours and safety concerns are primary barriers to children’s PA in early childhood [57] and independent active free play [58]. Moreover, two school-based reviews examining (a) factors that influence children’s active play [59], and (b) children’s perspectives on recess [35], reported key findings relating to safety concerns, rules and policies. Qualitative research that explores children’s experiences, attitudes, and motivations, can provide insight into an issue and improve understanding of health behaviours [60, 61]. Neither of the two previous school-based reviews were systematically conducted. Moreover, to our knowledge, no review has specifically examined children’s perspectives on safety and risk in active play. Therefore, this systematic review of qualitative literature aimed to synthesise research conducted with children that explored how safety and risk shape active play during recess in elementary and middle school. Specifically, this review sought to identify how risk and safety afford or constrain children’s play in schools, and how these factors serve to motivate or discourage children from playing actively. A secondary aim was to develop a model from the findings to guide efforts in schools to optimise children’s play opportunities during recess.

## Methods

This qualitative systematic review was undertaken according to the Preferred Reporting Items for Systematic Reviews and Meta-Analyses (PRISMA) checklist [62] and the Enhancing Transparency in Reporting the Synthesis of Qualitative Research (ENTREQ) statement [63] (Additional file 1). The review was prospectively registered with PROSPERO (CRD42021238719 Registered on 23/02/2021). The review began as a synthesis of qualitative research examining both child and adult perspectives and behaviour, and the methods described below reflect this. However, due to the amount and richness of the data generated from child perspectives alone, research conducted with children warranted its own review. Thus, this systematic review is concerned with research conducted with *children* to generate insights into their perspectives, experiences, and behaviour.

### Literature search strategy

A comprehensive and systematic search was undertaken across six bibliographic databases: Education Source, ERIC, MEDLINE, PsycINFO, SPORTDiscus, and Embase. A search strategy, which combined terms for ‘child’, ‘teacher’, ‘principal, ‘parent’, ‘school’, ‘active play’, and ‘recess’, was developed and adapted for each database. The search was restricted to English language articles published from 2000 onwards, to concentrate on contemporary research on children’s active play in schools since changing attitudes to safety and risk in children’s play have been documented [55, 64–66]. The final search was completed on 26/03/21. A full description of the search strategy and search terms are provided in Additional file 2.

### Study screening and selection

**Fig. 1:**
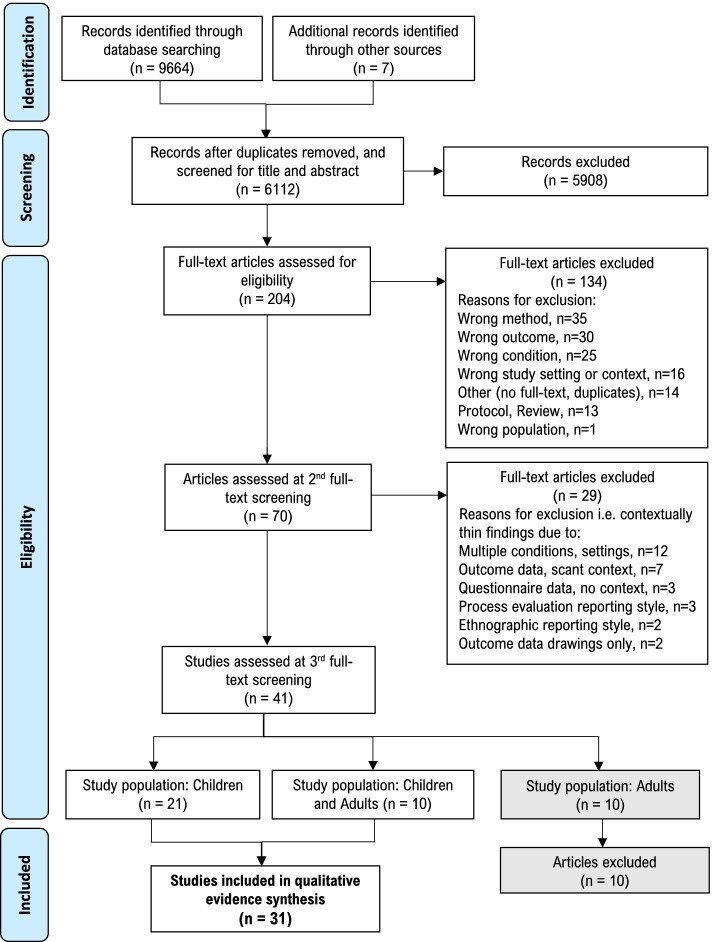
PRISMA flowchart

Search results were imported into Clarivate Analytics EndNote X9, duplicate records were removed, and remaining records imported into Covidence [67] for screening. Study screening and selection was undertaken in four stages. *First*, at title abstract screening, studies were required to meet the six eligibility criteria described in Table 1. *Second*, at the full-text screening stage, an additional ‘risk’ or ‘safety’ outcome criterion was applied for inclusion in the review (see Table 1 for definitions). However, the number of studies that met the eligibility criteria after full-text screening (*n* = 70) was considered too vast to conduct a meaningful comparison and analysis, so a further *third* stage of screening and selection was required [58]. This was conducted through a two-step process of (1) extracting the characteristics of studies included after full-text screening in a standardised Excel spreadsheet, and (2) re-screening against a further eligibility criterion of ‘contextually thick data’ (defined in Table 1). At all three stages, records were screened for eligibility independently by teams of two reviewers (AJ, EE, KF, LB, NL) and discrepancies were discussed by the review team until consensus was reached. Reasons for excluding articles at stages 2 and 3 are reported in Fig. 1. The number of studies meeting the eligibility criteria at the end of stage 3 (*n* = 41) was, again, considered too vast (based on the range of studies and volume of data) [58], and a final, *fourth* stage of screening was conducted to narrow the study population to children only (see Table 1 and Fig. 1).

**Table 1:** Eligibility criteria for inclusion of studies in the framework synthesis

	**Inclusion**	**Exclusion**
STAGE 1 CRITERIA: TITLE/ ABSTRACT SCREENING		
**Article type**	Original research published in peer-reviewed academic journals	Conceptual or theoretical papers, opinion pieces, reviews
**Population**	Typically developing children or early adolescents with a mean age between 5 and 14 years AND/OR Adults with a role relevant to children in the school setting (e.g. teachers, yard duty supervisors, school administrators, school nurses, parents). The aim of the research must be to explore adults’ behaviour and/or perceptions in relation to children’s active play and/or risky play in schools	Children older or younger than the age range specified Children with a medically diagnosed condition e.g. asthma, autism, epilepsy, intellectual disability, immune disorder etc Adults’ perceptions of PE, active lessons, structured recess or children’s active play or risky play outside of school
**Study setting**	Elementary or middle school (or equivalent) settings	Before- or after-school programs, early childhood programs, high schools
**Context**	Recess: defined as “*the non-curriculum time allocated by schools between lessons for youth to engage in leisure activities*” ([68], p.3)	Structured classroom activity breaks, active lessons, physical education classes, outdoor education programs, outdoor learning
**Condition**	Active play or risky play: Active play: defined as *“a form of gross motor or total body movement in which children exert energy in a freely chosen, fun, and unstructured manner”* ([17], p.164) Risky play: defined as *“thrilling and exciting forms of physical play that involve uncertainty and a risk of physical injury”* ([53], p.22) In recognition of the wide variation in the literature for terms pertaining to children’s play, the following alternative terms were included: outdoor play, free play, unstructured play, physical activity during play, unstructured physical activity, child play, challenging play, and adventurous play	Structured-play, structured-recess programs such as walking interventions, teacher-organised recess activities
**Research method**	Original research employing at least one qualitative research method such as focus groups, observation, or walking interviews Mixed methods studies were included if data from the qualitative components could be extracted and analysed independently of the quantitative results	Quantitative research methods e.g. experimental, quasi-experimental, cross-sectional and cohort studies
STAGE 2 CRITERIA: FULL-TEXT SCREENING		
**Risk or safety outcome**	Safety or risk-related finding or theme in relation to children’s active and/or risky play Risk: defined as *“the effect of uncertainty (whether positive or negative) on objectives”* [69] Safety: defined as *“a state in which hazards and conditions leading to physical, psychological or material harm are controlled in order to preserve the health and well-being of individuals and the community”* [70]. Notably this definition of safety includes safety from both physical and psychological harm	Study findings relating to safety and risk in schools that are not directly related to active play or risky play, such as: gun violence, soil or air pollution, microbial infections
STAGE 3 CRITERIA: 2^ND^ FULL-TEXT SCREENING		
**Outcome data is contextually thick**	Risk and safety findings must be contextually *thick* Contextually thick descriptions identify both an ‘issue’ (e.g. a risk or safety finding in play) and its context, and the context provides the social or cultural meaning to the issue, thereby aiding it’s symbolic importance and understanding [61]	Risk or safety findings are contextually *thin*, due to: 1. Scope: multiple conditions or setting domains investigated; 2. Outcome data reported too brief; 3. Method: Questionnaire within insufficient qualitative data; 4. Process evaluation reporting of intervention or outcomes with thinly described data; 5. Ethnographic reporting method where ‘findings’ cannot be differentiated from the remainder of the article; 6. Method: relevant data limited to children’s drawings without children's own explanation of meaning
STAGE 4 CRITERIA: 3^RD^ FULL-TEXT SCREENING		
**Population: children**	Children or early adolescents with a mean age between 5 and 14 years Studies where both children and adults were participants were included if data relating to child participants could be extracted and analysed independently of the adult participants	Adults with a role relevant to children in the school setting (e.g. teachers, yard duty supervisors, school administrators, school nurses, parents)

### Synthesis method

Congruent with best-practice recommendations for qualitative evidence synthesis [71], the synthesis approach was determined once all studies were included. Due to the number of studies that met the inclusion criteria and the breadth of research methods employed, framework synthesis was selected; a systematic but flexible method allowing both aggregation and configuration of findings [72]. In the context of this review, where the review questions were open and theory was emergent, analytic procedures were configurative, and the framework evolved during analysis to develop theory [72]. There are two key stages and five overlapping steps in framework synthesis as depicted in Table 2.

**Table 2:** Application of the framework synthesis method

Framework synthesis stage	Synthesis steps	Application in this review
***Stage 1*** *Developing an initial conceptual framework*	1.**Familiarization**: Becoming immersed in the data	Undertaken during full-text screening and study selection (both stages), in addition to reading quantitative literature, systematic and narrative reviews for the field, handsearching references
	2.**Framework selection**: Identification of key themes to inform the framework	Systematic extraction of salient themes and findings from 18 studies identified in Step 1, identification of relevant theory and definitions (see Additional file 3 for full description)
***Stage 2*** *Recognising patterns of data through an iterative process of aggregation and configuration*	3.**Indexing**: Systematically tagging and labelling key themes in the data	Data extracted, labelled, and indexed in NVivo software, using codebook developed from initial conceptual framework. Data not fitting framework analysed inductively
	4.**Charting**: Devising a series of thematic charts to allow the full pattern across papers to be explored and reviewed	Themes developed and revised iteratively in NVivo. Findings/ themes charted in Excel, patterns across data and studies explored
	5.**Mapping and interpretation**: Drawing together the synthesis, consideration of how the themes answer the review question	Conceptual framework developed further to reflect review findings. Relationships between themes mapped and illustrated in Figures using PowerPoint

The ‘Framework synthesis stage’ and ‘Synthesis steps’ columns are informed by the work of Brunton et al. [72] and Gough et al. [73].

### Initial conceptual framework and codebook development

The defining feature of framework synthesis is the development of an initial conceptual framework, and the emergent framework that is the outcome of the review [73]. As no existing framework was identified to guide the synthesis, a comprehensive and systematic approach was taken to develop the initial conceptual framework from the literature and a supporting codebook to guide data extraction [72, 73]. Structured on the socio-ecological model (SEM) [74] and underpinned by Gibson’s theory of affordances [75], the framework represents five levels of influence on children’s recess active play behaviour: individual, interpersonal, physical environment, policy and institutional, and societal. Affordances describe the ‘functional possibilities’ that the environment, and objects in the environment, can provide to an individual [76, 77]. In the context of active play, affordances provide children with opportunities to climb, run, jump, swing, balance etc. (See Table 3). Across the five SEM levels in the initial framework, 25 risk and safety themes were identified, which may ‘afford’ or ‘constrain’ active play in schools. The process undertaken to develop the initial framework, justification for the theories underpinning it, the resultant framework, and the codebook, are provided in Additional file 3.

### Data collection

Using a standardised data extraction Excel spreadsheet, developed by the primary investigator, two authors (AJ, KF) independently extracted study characteristics, including author, year, country, study design and theoretical framework, sampling methods, school setting and participant characteristics, qualitative data collection and analysis, and rigour (Additional file 4). Any discrepancies were discussed until consensus was reached. The rigour and methodological quality of each study was evaluated using the Critical Appraisal Skills Programme Qualitative Checklist (CASP Checklist) [78–80]. No study was excluded based on appraisal results, in recognition of the diversity in qualitative research approaches and reporting styles, which can influence appraisal outcomes, and therefore, potentially underrate or overrate the quality of an article [79, 81]. The CASP Checklist is comprised of two screening questions (pertaining to aims of study and appropriateness of qualitative methodology to aims) and eight appraisal questions (research design, recruitment strategy, data collection, reflexivity, ethical issues, rigour of data analysis, and the reporting and value of findings) [78]. Two authors (AJ, MD) independently appraised all eligible studies using Covidence software. Criteria for what constituted each answer option for each of the 10 CASP items were developed and agreed by MD and AJ. Disagreements in appraisal were discussed until consensus was reached, for example, assessment of item 6 (researcher positionality and potential for bias in the research process) was rarely explicitly addressed, and therefore, required interpretation between review authors.

Study results were taken to be anything labelled ‘results’ or ‘findings’, and consisted either of verbatim quotations from study participants or findings and observations reported by authors [73]. Quotations, author interpretations and observations were given equal weighting. For the studies that included both child and adult participants, only data relating to the child participants were extracted. Studies were imported into QSR NVivo software (version 1.5) to aid data management and analysis.

### Analysis and synthesis of results

Using the codebook developed for this review (Additional file 3), data identified as risk or safety findings were extracted, labelled, and indexed (coded) by one author (AJ). To enhance reliability of the synthesis, the codebook was tested by four authors (AJ, EE, LB, NL) with a subset of four studies (13%). Indexing between authors for each study were compared and discussed and the codebook refined. As described in Table 2, all data were initially labelled descriptively (indexed), and then analysed deductively (using the codebook) and inductively (e.g. where extracted data did not translate into any pre-existing themes), to develop new themes, consistent with thematic analysis [82]. Findings were then charted, mapped, and interpreted to identify patterns across data and studies, through a process of configuration [73]. This iterative process was not conducted linearly, but rather cyclically, whereby, themes evolved as more data was synthesised [72]. On completion of this process, a new framework emerged, which integrated the initial conceptual framework with the new concepts and themes [72].

### Positionality / reflexivity

Considering the findings of this review and how they were reached in the context of the researchers’ worldviews and background is important for transparency and trustworthiness [83]. This review adopts a critical realist epistemology which proposes that knowledge of reality is mediated by our perceptions and beliefs [76]. Authors in this review have backgrounds in education (EE, KF, NL), health promotion (AJ, LB), physical literacy (AJ, EE, KF, LB, NL, MD), public health (AJ, LB), qualitative research methods (AJ, EE, KF, LB, NL), sport science and motor skill development (EE, KF, LB, MD, NL), and systematic reviews (EE, LB, MD, NL). The authors met regularly throughout the review process to discuss the review stages, progress, and team reflections. In particular, the review team had many in-depth discussions about how risk and safety were studied and reported, what constituted a risk or safety finding, and the influence of differing epistemological perspectives [84].

## Results

### Study selection

The electronic database search identified a total of 9664 records. After three stages of detailed screening, a total of 41 studies met the criteria for inclusion in the framework synthesis. However, as detailed in the methods, the decision was made to split the review between children’s perspectives and behaviour, and that of adults, resulting in a final total of 31 studies included in this review. Figure 1 shows a PRISMA flow diagram of the screening process, including the pre-defined reasons studies were excluded at the 1^st^ and 2^nd^ full-text screening stages, and the split between child-based and adult-based research.

### Characteristics of included studies

Of the 31 studies included in the synthesis and described in Additional file 4, most were conducted in England (*n* = 8) [85–92], Australia (*n* = 7) [32, 93–98] and Denmark (*n* = 4) [99–102]. Three studies each were conducted in Canada [103–105] and the USA [106–108], and one study in each of Finland [109], Iceland [110], Netherlands [33], Spain [111], Sweden [112], and Tanzania [113]. A total of 15 studies focused on children’s physical activity during recess as the phenomena of interest [32, 33, 85, 87, 90, 93, 94, 97–100, 103, 104, 109, 111], while 10 studies were interested in children’s development and play more broadly [86, 88, 89, 91, 92, 95, 96, 101, 102, 105, 107, 113]. The remaining studies covered a range of other disciplines, including, environmental education and health (*n* = 2) [89, 110], education (*n* = 1) [108], injury prevention (*n* = 1) [112], psychology (*n* = 1) [106], and human geography (*n* = 1) [88].

Not all studies clearly specified the number of participants, particularly where school playground observation was employed, however, of reported data, we were able to estimate at least 1408 children across 140 schools participated in the studies. Most studies sampled an approximate 50:50 ratio of girls and boys, while two were conducted solely with girls [95, 100]. Children’s ages were reported inconsistently; most reported the study population as an age or grade range and few reported actual numbers of children by age. While all studies were conducted in elementary schools (generally entry-level to grade 6), the year and grade levels in these institutions did not always correlate and varied across jurisdictions, with some including up to Year 7 [32], or Year 8 [99, 104, 105], and one study including Year 7–8 participants who were aged 13–15 years (from one of five schools) [99]. Two studies included Year 7 participants from a secondary school [93, 94]. Most studies were conducted in either urban settings (*n* = 12) or combined urban and rural settings (*n* = 9), while only two studies were conducted solely in rural settings, and eight studies did not report setting location.

Although not consistently reported, a range of study designs and methodologies were employed, including participatory action research (*n* = 4) [33, 90, 106, 110], ethnography (*n* = 4) [88, 89, 91, 101], case study (*n* = 3) [90, 93, 109], formative, process, and outcome evaluations (*n* = 3) [99, 100, 108], qualitative descriptive (*n* = 2) [32, 97], explorative (*n* = 2) [85, 95], phenomenology (*n* = 1) [87], mosaic approach (framework of child-oriented methods) (*n* = 1) [86], observational (*n* = 1) [107], and field study (*n* = 1) [112] designs. Of the 31 studies, 18 employed methods to elicit children’s perceptions and experiences only, while 13 studies employed a mix of playground observation and methods to elicit children’s perspectives. The most common method for eliciting children’s perspectives were focus groups (*n* = 16), while 17 studies employed visual methods such as photo-elicitation (*n* = 8) and drawing (*n* = 9). The most common analysis technique was content analysis (*n* = 9) [32, 86, 96, 100, 103, 107, 109, 111, 112], followed by thematic analysis (*n* = 5) [85, 95, 99, 104, 110], ethnographic analysis (*n* = 3) [88, 89, 101], and interpretative phenomenological analysis (*n* = 1) [87].

### Quality appraisal

Most studies provided a clear statement of research aims and a research design that was appropriate to address these aims. Studies also generally provided a clear statement of research findings and discussed the contribution their study made. However, study methods were inconsistently reported, particularly recruitment methods, ethical considerations, and the analysis process. Thirteen studies did not describe a named qualitative analysis method, and many did not present disconfirming data or discuss how data presented were selected from the original sample. Of note, 71% of studies did not critically examine the relationship between the researcher and participants, the researcher’s positionality, or the potential for bias or influence during the research process. The quality appraisal results are provided in Additional file 5.

### Synthesis findings: risk and safety themes

**Fig. 2:**
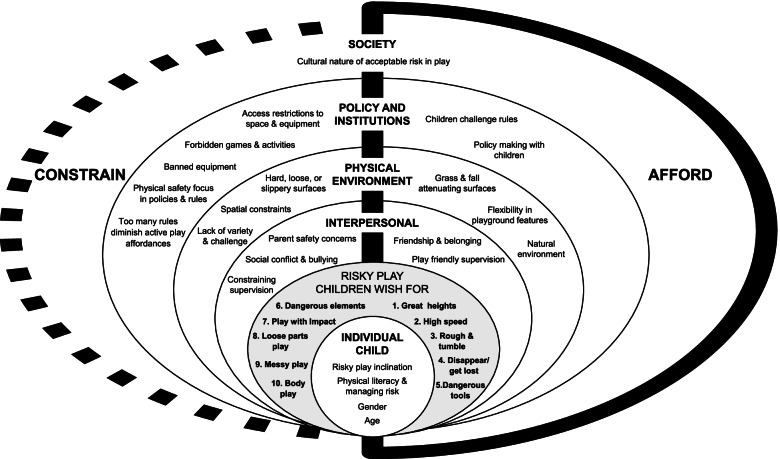
Socio-ecological model of risk and safety factors that shape children’s active play in schools. Legend: The socio-ecological model represents the emergent conceptual framework for risk and safety factors that shape children’s active play in schools across 5 SEM levels (Individual, Interpersonal, Physical Environment, Policy and Institutional, and Societal), together with 10 types of risky play children wish for in schools. The framework consists of 11 constraining factors, 7 affording factors, 4 factors that afford or constrain at the individual level, and 1 factor that affords or constrains at the societal level

The emergent conceptual framework is represented in the socio-ecological model in Fig. 2, depicting 23 risk and safety themes across five levels of the SEM that afford or constrain active play during recess, together with 10 types of risky play children wish for in schools, as identified through research conducted with children. Building on the initial conceptual framework (developed from research with children and adults), themes were adapted, some excluded, and six new themes were created. Each theme is described below. Where reported, children’s grade (G) or year (Y) level is described, as this was more consistently reported than age.

### Child characteristics

#### Risky play inclination

Across schools and jurisdictions, children expressed enjoyment of and a desire for risk-taking and challenge in play. Moreover, playground observations described risky play activities, even where such activities were not permitted. Table 3 details the 10 risky play types described and observed across studies. Fast ball games such as basketball, football, and soccer are included in the ‘high speeds’ category, although they often also involved rough and tumble play [86, 91, 111]. Despite being traditional sports games, they were categorised as risky play in this review where they met the risky play definition (See Table 1), were child-led, and involved flexible rules made up by children and adapted to their play environment. ‘Risky’ sports games were commonly restricted in schools due to safety concerns [92, 103, 104, 111]. Additionally, children’s desire for ‘body play’ (resistance activities that afforded tumbling, bouncing, balancing and pulling, and included gymnastics-like activities like cartwheels, somersaults, trampoline play and tug of war games [95, 100]) was common, especially for girls, although it was rarely accommodated in schools outside the Nordic region.

**Table 3:** Risky play children wish for in schools

Risky Play (RP) Type	Risk element	Play affordances	Example activities in schools	RP children enjoyed or desired	RP observed in school playgrounds
1.**GREAT HEIGHTS**	Danger of injury from falling	Climbing, jumping, balancing, swinging, hanging	Tree climbing Climbing equipment (e.g. monkey bars, climbing walls, low/high ropes course), Climbing non-play structures (e.g. fences, stairs) Stilts	[33, 85–88, 93–95, 99, 100, 103, 106, 110, 112]	[89, 110, 112]
2.**HIGH SPEED**	Uncontrolled speed and pace that can lead to a collision with something (or someone)	Running, swinging, sliding, sledding, cycling, skating, kicking, throwing, catching	Tag, British bulldogs, and other chasing games Scooters, skates, skateboards, bikes Swings, slides, slippery dip, flying fox, spinner Fast ball games like football, soccer, basketball (where child-led with rules adapted by children to play environment)	[33, 85–87, 90, 93–96, 99, 103, 104, 109–111]	[91, 96, 101, 106, 107, 110, 112, 113]
3.**ROUGH AND TUMBLE**	Children may harm themselves or each other	Play-fighting e.g. wrestling, fencing, Running and throwing (e.g. tagging with balls) Running and catching/ holding	Play-fighting, super-hero play, sword-fighting (with sticks), conkers Snow fights Ball-tag games (dodgeball, brandi, skobolti) Catch and contain games (fire-catch)	[33, 85, 87, 93, 94, 96, 97, 99, 101, 104, 106, 110, 112]	[89, 92, 96, 101, 105–107, 112]
4.**DISAPPEAR OR GET LOST**	Children are unsupervised, alone	Hiding, getting lost and found again	Hide and seek and other play in unsupervised or ‘out of bounds’ areas Playing in tree houses, bushes, trees, dens Mazes, tunnels	[32, 33, 86, 93, 97, 99, 100, 102, 109–111]	[88]
5.**DANGEROUS TOOLS**	Potential for injuries or wounds	Cutting, whittling, sawing, drilling, tying	Building dens with tools like hammers and saws Playing with (skipping) ropes in ways other than intended e.g. to make swings or tie children up in chase and catch games		[90, 105, 106]
6.**DANGEROUS ELEMENTS**	Risk of injury from falling into or from something	Sledding, sliding, skating	Playing near fire, deep water, frozen water, steep hills Sliding down snowbanks or on ice	[86, 104, 109, 110, 112]	[89, 112, 113]
7.**PLAY WITH IMPACT**	Risk of injury through impact	Running, pushing, pulling	Games that involve crashing and colliding Pushing and shoving in play (e.g., games like ‘hill’)	[94, 101, 110]	[101, 112]
8.**LOOSE PARTS**	Danger of injury from sharp or heavy objects. Use of dirty objects	Lifting, carrying, pulling, balancing, climbing	Den building in natural environments Loose parts play with re-purposed materials such as tyres, timber, milk crates, tarps	[33, 100, 110]	
9.**MESSY PLAY**	Danger of illness from unsanitary or cold environments	Digging, jumping, splashing, throwing, running, sliding	Sand pit, foam pit Water play, muddy puddle play, digging in dirt and gardens, snow and ice play	[85, 88, 93, 109, 110]	[89, 110, 113]
10.**BODY PLAY**	Children may harm themselves or each other by falling or colliding	Body inversion, tumbling, balancing, bouncing, pulling	Gymnastics-like activities e.g. cartwheels, somersaults, handstands Dance Trampolines, tumble bars Tug of war	[33, 86, 93–95, 99, 100]	[89, 106, 113]

The ‘Risky play type’, ‘Risk element’ and ‘Play affordances’ columns are informed by the work of Heft [114], Sandseter [64, 115], Kleppe et al. [116], and Jelleyman et al. [117].

Analysis of children’s discussions about active play revealed they were motivated to take risks and seek challenges in play for fun and enjoyment [32, 33, 90, 93, 94], thrill and excitement [93–95, 101, 112], physical challenge and competition [32, 86, 87, 95, 100, 110, 112], testing physical limits and playing on the edge between fear and exhilaration [94, 95, 110, 112], and mastering new skills [86, 93, 95]. For children these concepts were interconnected, for example, when children in an Australian study were asked to explain what fun and enjoyment in active play meant, they described it as being “*dangerous*” (boys) and “*challenging*” (girls) ([32], p.47).

#### Physical literacy and managing risk

A range of capacities and skills were described by children and observed in studies, that influenced their engagement with risk and how they kept themselves safe in the playground. As described in the codebook (and explained in Additional file 3), these capacities and skills were mapped to the definition (“*Physical literacy is lifelong holistic learning acquired and applied in movement and physical activity contexts*”, which integrates physical, psychological, social and cognitive capabilities) and four domains of the Australian Physical Literacy Framework ([118], p.5) (see Table 4).

**Table 4:** Physical literacy capacities and skills that influenced risk engagement and safety management in active play

Physical literacy domain	Capacities and skills	Children’s perceptions and experiences	Playground observations
Physical	Movement skills, strength, agility, coordination, fitness	[95, 101, 103, 112]	[91, 101, 112, 113]
Psychological	Confidence, enjoyment, self-regulation (emotions)	[32, 33, 93, 94, 103, 110, 112]	[101, 113]
Social	Relationships, cooperation	[95, 101, 105, 107, 108]	[91, 101, 105, 107, 113]
Cognitive	Safety and risk, rules, perceptual awareness	[86, 94, 96, 101, 112]	[91, 101, 112, 113]

The ‘Physical literacy domain’ and ‘Capacities and skills’ columns are informed by the ‘Domains’ and ‘Elements’, respectively, of the Australian Physical Literacy Framework [118].

Children described avoiding or limiting their risk-taking in play in response to perceived danger or awareness of their own physical limits. This was expressed as”*being careful*” [94, 96, 101, 112] or “*avoiding danger*” [86, 94]. Children identified physical environment features such as surfaces (concrete) or equipment (climbing frame), as well as other children (crowded areas, older children), as reasons to limit their play [33, 86, 96, 101, 103]. As a Y4 English child noted: “*I don’t really like the climbing frame because it’s really crowded, and they play lots of really weird and unsafe games and I never go on it*” ([86], p.1372). Conversely, other children did not fear injuries in play [93, 94, 101, 112]. As a G7 Australian child explained: “*I like hanging in trees…and on the flying fox…it’s fun falling off*” ([94], p.68). An ethnographic study that examined Danish children’s risk engagement over eight months, observed children’s willingness to take risks differed according to their physical skills and ability, with lower skilled children perceiving greater risks in games or activities that higher skilled children enjoyed without fear [101]. Across studies, children described ways they kept themselves safe: some practiced avoidance [32, 86, 96, 103, 111, 112], while others negotiated rules and conditions that enabled them to play in ways they were comfortable with [32]. Similarly, playground observations revealed lower-skilled children managed risk through avoidance [112] and the negotiation of conditions for safer play [91, 101].

#### Gender

Gender differences in risk-taking in play varied across schools and jurisdictions, and studies did not always report findings by gender. The most common types of risky play girls described enjoyment of, or desire for, were ‘great heights’, ‘body play’, and ‘disappear or get lost’. While boys frequently described ‘rough and tumble’, ‘high speeds’, and ‘great heights’ [86, 87, 91, 96, 99, 101, 112]. Boys were also observed by both researchers [101] and children [112] to take more physical risks in play generally. However, other studies indicated these differences may be nuanced, for example, the type of high speed or rough and tumble play influenced participation, and activities such as snow fights or chase and catch games appealed to children of all genders [33, 85, 87, 93, 104, 109, 110]. Moreover, the dominance of fast ball games limited the play affordances available to girls during recess. Girls gave several reasons for this, including being actively excluded from games; indirectly excluded e.g., boys not passing them the ball; perceptions of gender roles that precluded girls and boys playing together; and ball games taking up playground space, thereby relegating other children to the periphery (see theme ‘spatial constraints’) [33, 86, 96, 111]. Playground observations confirmed girls’ affordances for play were limited in this way [88, 96, 107]. Two girls from a rural Australian school illustrated this: “*sometimes at the oval it can be really horrible, boys are running all over the place, kicking balls so we have to be stuck in a corner*” (Y4 girl), “*we could play dodge ball or tip at the oval but you have to be careful you could be bumped over and get hurt*” (Y2 girl) ([96], p.499).

#### Age

Age-related differences in risk-taking in play were limited, however, this may be due to the variability in how age-ranges were sampled across studies and the inconsistency in reporting study findings by age. In some schools, older children reported a lack of age-appropriate challenge in playground equipment, which discouraged them from playing actively [33, 93, 100], while younger children identified older children as a potential cause of bullying or playground injuries, which could have the effect of constraining their play [33, 93–96]. This was often related to children’s perception of spatial constraints, for example, a G3/4 Australian child described her desire for a larger playground: “*Bigger ‘cause…year sixers…they bulldoze people sometimes and people fall over*” ([95], p.154). Like gender, playground observations confirmed that younger children’s play affordances were constrained by the dominance of fast ball games [96, 107]. Some schools dealt with these issues by segregating the playground by age group (see theme ‘access restrictions to space and equipment’), however, children were dissatisfied with these rules when they perceived their play was unfairly constrained [33, 88, 98, 103]. As a G5/6 Canadian child revealed: *“[If you're aged] 12 and over, you can't play [on the equipment] … I don't like that!*” ([103], p.437).

### Interpersonal factors

#### Parental safety concerns

Safety concerns of parents were described by children in two Australian studies. Children perceived parents’ safety concerns limited the kind of play affordances provided by schools [95] and influenced children’s behaviour in relation to where and how they played in the school yard, as a G7 Australian child explained: “*My mum doesn’t want me hanging out near the fence because I could get stolen*” ([94], p.72). Conversely, an elementary student perceived schools were more concerned with children’s safety than parents: “*Your parents aren’t really concerned of your safety as much as the teachers*” ([94], p.70).

#### Social conflict and bullying

Social conflict and bullying were common themes, although the distinction between the two concepts was not clear-cut. As authors of one study observed, “*students generally used the term ‘bullying’ quite loosely, therefore the definition may vary and include a continuum of social conflict*” ([105], p.12). Bullying and teasing were commonly described by children as a barrier to active play [32, 33, 86, 93, 94, 97, 105, 109]. Both bullying and conflict led to children feeling socially excluded or unsafe in the playground, which constrained their play opportunities [32, 33, 86, 93, 94, 96, 105, 109]. As an Australian child described: *“[Children] try and hide from the bullies so they can’t do much playing*” ([32], p.47). While Canadian children expressed: “*I wish there was less bullying and exclusion.*” “*Some kids get real aggressive when the teacher is not looking…*.”, “*I wish everyone would get along*.” ([105], p.12).

According to both children and playground observers, competition over play space and equipment was the most common cause for conflict, which often led to disputes over territory, arguments, physical fights, and sometimes injuries [33, 88, 89, 93, 94, 96, 97, 105–108, 111]. As American children explained: “*people fight over stuff like jump ropes”, “some people fight over balls,’’* and conversely, *“there’s not fighting when everyone’s playing*” ([106], p.132). While a G6 Canadian child, explained: “*I think that there is not enough equipment because there is a lot of kids in the school, and everyone just takes it all. And then the bullying starts*” ([105], p.15). Children also identified that conflict and bullying were triggered by a lack of things to do [94, 97, 105, 106]. Despite this, children and playground observations revealed schools commonly dealt with social conflict by constraining children’s play affordances further through equipment removal, banned games, and restricted access to space or equipment, which frustrated children, and could have the effect of exacerbating social and behavioural problems [88, 92, 104–106, 111, 112] As a G5 Canadian child noted: “*we can’t play football now because people were fighting and [pause], and, like, [there is now] nothing to do*” ([104], p.6), while an Australian child explained: *“…with a boring space…people get really mean and stuff…use equipment the way they aren’t meant to*” ([94], p.67).

Children’s skills for resolving conflict varied across and within schools. Playground observations revealed some children were able to negotiate and resolve playground disagreements swiftly [101, 106, 107], as an American child illustrated by suggesting to another child: “*let’s do rock, paper, scissors*” ([107], p.6). Other children had difficulty navigating disagreements, with some suggesting teachers and playground supervisors could play a role in helping them practice these skills [33, 94, 105, 107, 108]. Children in a Canadian study perceived conflict management to be an important life skill they needed the opportunity to learn, as a G5 child explained: “*so, like you could probably bring back foursquare [competitive schoolyard ball game that involves bouncing a ball between quadrants to opposing players], even though there are some poor sports, umm, but there are poor sports in life, so you need to deal with it.*” ([104], p.6).

#### Friendship & belonging

Feeling socially safe in the school playground was an important facilitator of active play. For children, this meant a sense of belonging and having someone to play with [32, 97, 109], no fights or exclusion in the playground [33, 109], and having the support of friends when needed [94, 105]. A G3 Finnish child emphasised the importance of friends for play: “*If someone was left alone. You can do almost nothing if you’re alone*” ([109], p.417). While a Canadian child revealed: *“[you] feel comfortable and safe at recess because you have friends around to help you when you need help, they will defend you as much as you will to them*” ([105], p.12).

#### Constraining supervision

Children described a spectrum of supervision practices that constrained their active play, from unengaged supervisors who failed to observe social conflict in the playground [33, 86, 105, 107], through to over-controlling supervision styles, whereby adults were focussed on safety and enforcement of rules at the expense of active play [32, 33, 88, 89, 93, 94, 106, 108, 109, 112]. Such practices were characterised by commands like “*Don’t run!”* [109], “*Climb down!*” [112], “*Don’t walk up the slides*” [106], “*Don’t play here, go over there!*” [89], which had the common effect of limiting children’s affordances, freedom, and agency in play. As Australian children in two studies expressed: “*All the fun stuff, the teachers say’that’s dangerous. You’re not allowed to do that’*” ([32], p.47), and *“…if there was too many teachers around, you wouldn’t be able to do anything, so it would be boring*” ([93], p.10).

Negative supervision practices such as teachers exhibiting threatening behaviour or disproportionate sanctions for classroom or playground rule violations, were also described by children, and observed in studies [33, 88, 90, 94, 103, 105, 107]. One such sanction was withdrawal of all play affordances – either equipment or permission to play, sometime for the duration of recess [32, 33, 88, 90, 98, 103, 105, 107, 111]. In some cases, children explained that rules were not communicated in a friendly way, for example, a Dutch child noted: “*the supervisors should be less strict, we think, because they get angry very easily and get tough*” ([33], p.11).

#### Play friendly supervision

Conversely, children described positive supervision practices that afforded active play while also maintaining safety. These ranged from supervisors participating in play and games with children [32, 33, 85, 87, 93, 97, 109], to supervisors being in the background but engaged and available to step in to prevent injuries or provide help if necessary [33, 85, 93, 94, 108, 110, 111]. Children also described the role supervisors played in addressing social conflict and inappropriate behaviour [33, 85, 108]. As Y4 English child explained, the supervisor’s role was *“[to stop children] from being mean to each other*” ([85], p.445).

### Physical environment

#### Spatial constraints

Children frequently described spatial constraints as a barrier to active play [32, 33, 88, 93, 95, 96, 109, 111]. For example, playgrounds that were crowded with limited free space, or border fences and structures between play areas, discouraged children from playing running and chasing games due to a perceived risk of injury from falling over or property damage, like broken windows from ball games [32, 33, 93, 95, 96, 109]. Playground size and the potential for injuries was also identified as the cause of restrictive playground rules such as ‘no running’ or forbidden ball games [88, 95, 96, 111]. When asked what they wanted in a playground, a G3/4 Australian child expressed: “*bigger because you aren’t allowed to run ‘cause it is too small*” ([95], p.154).

Playground observations and conversations with children revealed that perceptions of insufficient space or poorly designed playgrounds and insufficient equipment, also resulted in some groups of children monopolising play affordances [33, 86, 88, 95, 96, 105–107, 111]. For example, fast ball games dominated the open playground space in many schools, which favoured boys’ (especially higher-skilled boys), play at the expense of girls’ (and lower-skilled boys) activities [33, 86, 91, 96, 111]. Once confined to a certain area of the playground, children’s active play was constrained by the physical affordances available, and the rules that governed that space. A G1 Australian child explained the conundrum of being confined to a certain part of the playground (“the pebbles”) by ball games that dominated most of the available space: “*Tip [a chase and catch game] is fun, if it were safe and we were allowed we could play tip here, we could also play hide-and-go, but you also need somewhere to hide when you play hide-and-go…there is nowhere to hide in the pebbles, so we can’t play that either*” ([96], p.499).

#### Hard, loose, or slippery surfaces

The assessment of playground surfaces and the likelihood of injury from a fall were common themes in children’s discussions about the suitability of the playground for active play. Children identified hard, slippery, or loose surfaces such as asphalt, concrete, gravel, or stones, as barriers to running and chasing games [32, 33, 86, 93, 95, 96, 98, 100, 110]. This was explained by a Dutch child, who revealed: “*well look, you have, say, that gravel, and when you fall, say, little pieces of gravel cut into your hand*” ([33], p.10). Similarly, an Australian child explained: *“… it’s dangerous at the pebbles, you can’t run on there, you could easily fall, one day a girl in our class fell and started bleeding*” ([96], p.499). Safety concerns associated with playground maintenance were another barrier to active play described by children, and observed in studies, most commonly in relation to surfaces [32, 33, 85, 94, 96, 106, 107, 109]. For example, slipping or tripping hazards [85, 96, 109], and hygiene, such as litter [33, 94, 106].

#### Grass and fall attenuating surfaces

In contrast, children preferred grass or synthetic grass for its softness and injury protection from falls [86, 94–96, 98, 100, 110], as illustrated by a G1 Australian child who said, “*me and my friends like chasing and racing each other, sometimes we fall and nobody gets hurt or cries because the ground is covered in grass, so you don’t get hurt*” ([96], p.501). This was reiterated by another Australian child, who explained “*you don’t get hurt [on grass] the only thing that happens is you get muddy*” ([98], p.213). Additionally, children desired fall-attenuating surfaces around playground equipment, such as rubber tiles, or wood-chips, which were lacking in some playgrounds [33, 86, 94, 96], or suggested protective equipment like helmets and knee pads to improve play safety [94].

#### Lack of variety and challenge

Equipment in the playground played a central role in children’s active play. Primarily, children desired more variety and challenge in play affordances, in relation to both fixed structures and loose equipment [33, 93, 95, 98–100, 103, 109, 110]. Lack of sufficient variety and challenge often led to existing equipment being used in ways other than intended, sometimes inappropriately [33, 87, 88, 90, 94, 95, 97, 100, 105, 106, 109]. As a G5 Australian child explained: “*people don’t use the equipment right when it’s boring…they can just sort of hurt someone whey they are bored…[and] make things destructive*” [94, p.68]. And another child explained: “*They don’t put nets in the tennis courts because people will run into them and get hurt. They bounce off and it makes you faster*” ([94], p.73). Additionally, children used non-play features, such as lampposts, stairs, walls, fences, railings, and benches as alternative and challenging play affordances, which frequently contravened playground rules [33, 88–91, 106].

#### Flexibility in playground features

Children expanded their play repertoire through creative use of playground features and equipment to keep play challenging and interesting, and preferred features that facilitated this flexibility [33, 87, 95, 106, 109]. However, this could lead to children using equipment in ways other than intended by adults. This was illustrated through children’s play with skipping ropes, as a Dutch child explained: “*Look, those children are skipping. But when you do that for a very long time, it becomes boring. Don’t you ever have that? That when you do something so often, it becomes boring?*” ([33], p.12). While playground observations in other schools revealed that when children expanded their skipping rope play by creating new games, such as tug of war [106], and chase, catch and tie up play [90, 105, 106], such activities were constrained by supervisors on safety grounds. Another way children kept their play interesting was by creating competitive games, as an English child explained: “*I like to climb on the climbing frame because it’s so high and I like to race down with my friends*” ([87], p.93). Likewise, a Dutch child described a competitive game her friends had created: “*In the sandpit, with the sandpit as starting point, who can run the fastest and then you have to jump over the sandpit and then there are nets you have to go underneath*” ([33], p.7).

#### Natural environments

Natural features in the playground were valued by children for the risky play affordances they provided, including woodlands, bushes, and gardens for running, chasing, hiding, and disappearing games, and trees for climbing and swinging from [33, 94, 99, 100, 102, 109, 110, 112]. Children described the explorative and creative play affordances they enjoyed in nature, including sand, mud and water play, snow and ice play, and den building with tree branches and sticks [33, 86, 89, 100, 104, 109, 110].

Children also valued the open-ended play opportunities found in nature, as a Danish child described:

“*I really liked it before [the playground renovation]... We played war games all the time up in the woodland. We found sticks we used as machine guns, and we lay hidden in the edge of the woodland… There were not so many things there, so you had to make-up things yourself… It was just a big area where you could run around doing everything imaginable*” ([99], p.669).

Conversely, some children described fearing the natural environment, such as the risk of splinters in fingers from wooden equipment [33], and a preference for metal structures for safer play [98].

### Policy & institutions

#### Access restrictions to space and equipment

School policies and rules governing ‘access’ were widely reported and observed to constrain active play. These included out of bounds or forbidden areas of the playground or school grounds [32, 33, 88, 89, 93, 99, 102, 103, 106, 107, 109, 111] and facilities, sports or play equipment that children were not permitted to use during recess [32, 93, 103, 105–107, 109, 111]. As a G3 Finnish child explained, “*that locked door bothers, you cannot go there. And there is all the equipment*” ([109], p.418). Children perceived rules that restricted access to playground space limited active play affordances, especially, high speeds (e.g., chase and catch games) [93, 96, 106, 109, 111], play where children can disappear (e.g. hide and seek, den building)[99, 102], and messy play (e.g. access to grassed areas in winter) [88, 89, 91, 107, 111].

Sometimes access restrictions were age or grade-level based, such as segregated playgrounds or rotations on equipment and space, which some children perceived limited their play affordances [33, 98, 103]. As a Dutch child explained: “*the playground for the younger children is lots of fun and you can do all kinds of things there, but we’re not allowed to go there*.” ([33], p.11). Conversely, children who desired more playground space suggested grade-level access rules as a potential solution to over-crowding [33, 94]. As a G6 Australian child proposed: “*I would actually make it so that there’s a grade 6 playground, instead of a 5/6 playground so that there’s more room to play*” ([94], p.74). Another way children’s access to playground space and equipment was constrained was through weather or seasonal polices, such as indoor recess when it was too hot, cold, wet, or icy, outside [32, 88, 97, 103, 107, 111]. Children were often dissatisfied with these rules, as a G5/6 Canadian child explained: “*if it's too cold, you have to stay in. And I don't really like indoor recesses… 'Cause you can't run or do anything.*” ([103], p.437). This extended to the activities and games children were allowed to play in certain weather conditions, as illustrated by a G5 Canadian child from another study: “*We have pretty much not very many options to do in winter because we can’t throw snowballs, can’t slide on ice, and I can see why but maybe more wintery activities*” ([104], p.6).

#### Forbidden games and activities

Across schools, children described a wide range of activities and games that were forbidden during recess. Most commonly, ‘rough and tumble’, ‘high speeds’, and ‘great heights’ were restricted or prohibited. Examples included British bulldogs [85, 92], dodgeball [105, 106], fast ball games like football or baseball [92, 103, 104, 111], wrestling or play-fighting [92, 112], tree climbing [33, 88, 94], snow and ice play [89, 104, 106], and in one school, skipping [92]. Sometimes games were restricted to certain parts of the playground, which children perceived led to unequal access to play affordances [93, 94, 96, 111]. According to children, games and activities were banned for a range of reasons, including perceived danger and risk of injury [32, 88, 89, 106], the belief activities or games were too aggressive [85, 112], or as a standard response to playground injuries or conflict [88, 92, 104, 106, 111, 112]. As a Spanish child explained: “*previously teachers let us play football, but because some guys were throwing the ball so hard and hit others…and they fall down*” ([111], p.4).

#### Banned equipment

Across schools’, children described equipment that had been removed or banned due to previous injuries or the potential risk of injury. This included fixed structures like swings or ziplines [103, 112], and loose equipment because it was used in an inappropriate manner, for example, skipping ropes used to tie up ‘villains’ in role-playing games [105, 106]. An extreme example of this was an English school that did not have any fixed or portable equipment, which the children explained was “*because of health and safety reasons*” ([87], p.92).

#### Physical safety focus in policies and rules

A recurring theme among children was the way playground rules and restrictions reflected an overriding concern with ‘physical safety’, which came at the expense of fun active play affordances, and sometimes social and emotional safety [32, 33, 87, 88, 93, 94, 98, 103, 104, 108, 109, 112]. A Canadian child emphasised this: “*It [would be easier for students to be active at school] like, if it were less stricter*” ([103], p.438). While a Y6 Australian child explained: “*fun spaces aren’t in schools…the safer ones [spaces] have to be in schools, because it’s the teacher’s responsibility*” ([93], p.9), and a Dutch child observed: “*the rules that are really necessary [about social manners] are hardly paid attention to*” ([33], p.12). Children perceived adults’ safety concerns to be the reason behind banned games, activities and equipment, and access restrictions to playground space and equipment [32, 33, 85, 93, 94, 103, 105, 106]. This was confirmed by researcher observations [89, 92, 97, 109, 112], as a Swedish researcher explained: “*children believe that many of their games are limited because it looks dangerous, but which according to them it’s not, because they are used to playing that way*” ([112], p.6). Moreover, a Finnish researcher described children’s perspective on authority as “*limiting children’s ability to implement their own ideas as to how to be active… [with supervisors] …controlling children’s actions, even if they were acceptable from a safety point of view*” ([109], p.417).

In some schools, children perceived that playground supervisors appeared to have their ‘own’ rules [33, 88, 106] or interpreted and applied rules inconsistently, leading children to question their basis [33, 88, 90, 103, 112], describing them as “*unfair*” [88, 103] or”*stupid*” [33]. Although children understood the safety rationale for rules (e.g., limiting fast ball games like soccer to certain areas), they felt they could be too wide-ranging, which limited children’s affordances for active play unnecessarily (e.g., banning all ball play in other areas, even soft balls) [32, 33, 98, 103, 104, 106]. As a Dutch child explained: “*They should let you do more. (…) It could be a nice playground if you could do more. There are some fun things, but you’re not allowed to play with them*” ([33], p.11). According to this student, the supervisors’ attitude was: “*There should be zero risk that you fall or get hurt!*” ([33], p.11).

#### Too many rules diminish play affordances

There was no one rule or policy, universally experienced across studies that restricted active play, however, the sheer number of rules and restrictions children experienced during recess was consistently reported across jurisdictions and schools. As an English child declared: “*We’ve got a lot of rules. We have got a page full of rules. Mr J–- [headmaster] tells us the rules. He says, don’t do this don’t do that …*” ([89], p.493). The multitude of rules and restrictions in schools could have the effect of substantially diminishing children’s affordances, freedom, and agency in play. As a G3 Finnish child noted: *“it is not nice, when they are controlling us all the time about how we go and what exciting things we do at recess”* ([109], p.417). For children, the combined effect of playground rules and controlling supervision, could render recess “*boring*” [33, 93, 94, 103, 106], and “*not fun*” [32, 33]. As a G7 Australian child explained: *“…if you take the tackling out of football… that becomes boring*” ([94], p.73).

#### Children challenge rules

Across schools, children reported and were observed challenging adult authority by breaking rules they didn’t agree with [33, 88–90, 92, 94, 102, 103, 106]. This was illustrated by Danish children, who revealed: “*We are not allowed to go into the bushes, but we do it all the same when the teacher on playground duty is not looking*.” ([102], p.168). Likewise, a G5/6 Canadian child declared: “*I would make our playground bigger. And I'd break the rule for letting everybody go on it. 'Cause apparently we're not allowed to go on it, but sometimes I still do.*” ([103], p.438). While a G7 Australian child explained: *“…no matter what…if people are bored…people are going to break the rules and do what they want”* ([94], p.67). Playground observations reiterated this sentiment [88–90, 106], as described by an English researcher: “*I saw lots of tennis balls being used for football, despite the ban, and a boy swing a rope around in a way that was clearly different to its intended use!*” ([90], p.365). Children also challenged adult authority by breaking playground rules in subtle ways, as illustrated by English children who were observed resisting the rule of ‘keeping off the grassed sports field’ by walking with one foot on the grass and one foot on the tarmac [88]. Children described taking pleasure in breaking rules, through which, researchers observed, children demonstrated agency and challenged adult authority [88, 90].

#### Policy making with children

Children wanted to be consulted on playground policies and rules and had many suggestions for improving active play affordances through playground policies and rules. These included positive rules like”*Only for running!* “ and”*Jump here!* “ [109], nuanced rules rather than blanket-ban rules [33, 89], designated play areas or staggered recess for different age groups [33, 94, 96, 105], and prioritising social wellbeing through enforcement of rules for social manners [33, 105, 106]. Overall, fewer restrictions and a decreased focus on safety was suggested by children across jurisdictions and schools [33, 89, 94, 103, 104, 106, 108, 109]. As a G5/6 Canadian child declared: “*And bring the safety level down, because we're not allowed to play badminton outside because we might get a birdie in the eye… That's life people! I think we should bring the safety level down a notch at least*.” [103, p.438].

### Society

#### Cultural nature of acceptable risk in play

Playground observations and conversations with children revealed all 10 risky play types (see Table 3) were frequently prohibited, although this varied across schools and jurisdictions. For example, Nordic region children enjoyed forms of play at high speed (e.g. skating, cycling, scooter riding) and rough and tumble (e.g. snowball fights, playfighting) to varying degrees [99–101, 109, 110, 112], whereas these activities were prohibited in English, Canadian and American schools [88, 92, 104, 106]. Conversely, in some Swedish schools, the rough and tumble game ‘king of the hill’ was banned [112], while Danish children were permitted to play their version of ‘hill’ on an asphalt playground [101]. Some Dutch and Australian children [33, 94] wished they could climb trees at school, whereas in Nordic schools, this was a regular playground activity [100, 110, 112]. Additionally, the belief children shouldn’t get cold, wet or muddy during recess, shaped restrictive playground policy and rules in Canadian, English, Spanish and American schools [88, 89, 91, 103, 104, 107, 111], whereas snow play and/ or messy water play was accommodated in Icelandic, Finnish, and Tanzanian schools [109, 110, 113].

## Discussion

This is the first systematic review of qualitative research to examine children’s perspectives on safety and risk in active play in schools. A key finding is that socio-cultural factors in schools, including the role of peers, supervisors, school rules, and cultural attitudes, have a substantial influence on children’s active play. Socio-cultural influences shape both the physical play environment (e.g., what equipment and features are provided), and how children play (e.g., what play is encouraged, permitted, or not permitted). Across SEM levels, themes were interrelated, indicating that constraints at one level influenced the play possibilities and constraints at other SEM levels [119]. Moreover, there were commonalities as well as conflicting perspectives among children across and within schools, highlighting diversity in children’s experiences and preferences. These findings are discussed in detail below, and a model for risk tolerance in children’s active play is proposed (see Fig. 4). Taken together, findings may inform future efforts to address the challenges in effectiveness, equity and sustainability of school-based PA interventions identified in systematic reviews of quantitative literature [31, 34], and present key messages for schools, and those who manage school policy, to harness play effectively for child benefit.

### Individual: child characteristics and risk engagement

Although only one study specifically explored children’s perceptions of risk-taking in play in schools [101], 10 risky play types enjoyed or desired by children in schools were identified in the synthesis (see Table 3). Similar to findings in early childhood settings [120], the most frequently depicted and observed risky play types were ‘great heights’, ‘high speed’ and ‘rough and tumble’. These were also the types of play that generated the most safety restrictions in schools.

Notably, our findings indicate children varied in their attitude to risk-taking in play and what was perceived as risky for one child was not necessarily risky for another. This is consistent with observational research of children’s play in early childhood settings [53, 64], and affordance theory [75]. Children’s ability to perceive affordances develops systematically as they grow and learn new skills [121]. In the context of active play, each child’s affordances are opportunities for behaviour that combine the objective nature of the environment (e.g. playground equipment, rules, other children) with subjective capabilities of the child (physical, psychological, social and cognitive); in other words, their physical literacy [115, 122]. Findings illustrate several ways children’s physical literacy influenced their risk engagement and safety management (see Table 4). Importantly, because active play also develops children’s physical literacy [123], a reciprocal relationship exists between them, i.e., the more a child plays actively, the greater their physical literacy [39, 101]. Conversely, the less a child plays actively, the less effective their physical literacy development and ability to avoid injury [51]. Over the longer term, limiting children’s exposure to risk in play may lead to other negative outcomes; psychologists contend children need opportunities to experience risk to develop the ability to cope with uncertainty and fear, without which, psychopathology disorders, such as anxiety, may result [39, 124].

The finding regarding physical literacy has important implications for policy and equipment decisions in schools, as a risk-averse approach may have the unintended effect of exacerbating safety issues and injury risks in the playground, disadvantaging children’s development over time [41]. Moreover, the relativity of risk between children indicates a variety of equipment and playground features is required to meet the play needs of all children. The dissatisfaction children described with the lack of variety and challenge in playground equipment underlines this need. Although research in early childhood settings has reported a significant positive association between children’s exposure to risk in natural play environments and their movement skills (an important element of physical literacy) [125], the relationship between risk-taking in play and physical literacy, using a wider definition, and in older children, is yet to be explored.

### Interpersonal & physical environment: equipment and space constraints, conflict, and access to play affordances

There were interactions at the interpersonal and physical environment levels between equipment and space constraints, access to play affordances, and social conflict. This is consistent with a meta-study that examined children’s perspectives on school recess [35]. According to Kytta [121], children’s ability to perceive and use affordances for play is regulated through fields of promoted action (e.g. culturally defined and socially approved affordances, such as the equipment provided and playground rules) and constrained action (e.g. affordances constrained by others, including peers, or inherently through their design, such as playground layout). Our findings suggest that the causes and effects of social conflict during recess were complex: children’s freedom and agency in play was constrained by the physical features of the play environment and competition for access to equipment and space, which was often further restricted by playground rules. Learning to maintain respectful relationships, including conflict resolution, is an important skill, which may help children navigate these issues, and as such, is another element of children’s physical literacy (in the social domain) that could be prioritised in schools [118].

Our findings also indicate that organisation and design of the playground (e.g., with open spaces that facilitate supervision) and dominance of traditional sports games in schools reflects how play is culturally defined, and shapes who gets access to play resources [86, 111, 126]. Research exploring gender socialisation and play in schools reported similar findings, highlighting the value of spatial analysis of schoolgrounds for active play research [77, 127, 128]. Moreover, Australian research found children, especially girls, value ‘in-between-spaces’ like small enclosures, edges and natural settings in schoolyards, despite these places often being out of bounds or overlooked by school authorities [129]. While Danish researchers have drawn the distinction between ‘children’s places’ and ‘places for children’, highlighting how school playgrounds, designed with safety and supervision in mind, often fail to adequately consider children’s play preferences [99, 102]. The element ‘connection to place’ in the psychological domain of the Australian physical literacy framework, describes this as children’s “*appreciation and connection to the environment…in relation to movement and PA*” ([118], p.35). Consideration of children’s connection to existing school playground features, and the gendered dynamics of access to play affordances more broadly, may require greater attention in playground interventions [99, 128]. Moreover, investigation of a wider range of ways children may experience unequal access to play affordances, e.g., based on ethnicity/race or socioeconomic status (SES), was absent in included studies.

### Policy & institutions: cycle of risk-averse decision making

**Fig. 3:**
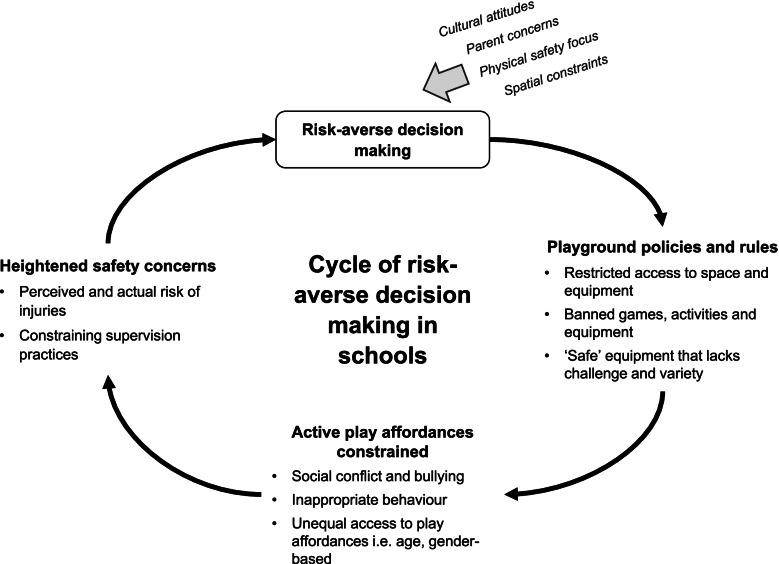
Cycle of risk-averse decision making in schools

Themes at the policy and institutional level concerned the nature and impact of rules in the school playground, which interacted across SEM levels to constrain children’s active play. Children perceived these rules represented adults’ concern with physical safety, which is consistent with the wider qualitative literature examining determinants of children’s PA behaviour in contemporary Western societies [57, 58]. A novel finding in the current review is the extent to which children will challenge adult authority and break rules they don’t agree with in school settings and how this can contribute to a cycle of risk-averse decision making. Too many rules and restrictions during recess reduced children’s play affordances, rendering recess ‘boring’, which contributed to social conflict, inappropriate behaviour, and unequal access to play affordances. This, in turn, could have the effect of heightening safety concerns and perceived and actual injury risks in schools, leading to further risk-averse decision making in the form of more rules and restrictions (see Fig. 3) [33, 88, 89, 92, 103, 105, 107, 111].

Evidence regarding the drivers for the physical safety focus in policies and rules was limited to children’s perceptions, but may include parental safety concerns, spatial or environmental constraints, and the cultural nature of acceptable risk in play. Wider research points to socio-cultural and economic factors in contemporary societies [41, 51]. For example, research with adults in schools and early childhood settings indicates duty of care policies and perceived litigation risk weigh heavily on teachers and administrators, while a lack of policy to promote active play and PA in schools, as well as a lack of children’s perspectives in policy, means quality play experiences are not prioritised [130–133].

### Societal: cultural attitudes towards acceptable risk in play

At the societal level, findings reveal that what constituted ‘socially approved’ play and play equipment varied across jurisdictions. Research in early childhood settings has reported similar variation, with Nordic countries, which traditionally place a high priority on children’s play outdoors [115, 134], more accommodating of risky play than other Western nations, such as Australia [131], Greece or Portugal [54]. Concerningly, however, there are signs the West’s preoccupation with safety may be negatively influencing Norwegian culture and practice around children’s play outdoors [135]. This shift also points to an opportunity to reverse the trend, as cultural attitudes are potentially malleable to change. A child-rights approach, based on the principles embedded in Article 31 of the CRC may provide a practical means to achieve this [28]. The UN Committee on the Rights of the Child identified the need to balance risk and safety in children’s play as a key challenge to be addressed in the realisation of Article 31, and recommended ”*the best interest of the child, and listening to children’s experiences and concerns, should be mediating principles for determining the level of risk to which children can be exposed*” ([12], p.12).

### Model for risk tolerance

**Fig. 4:**
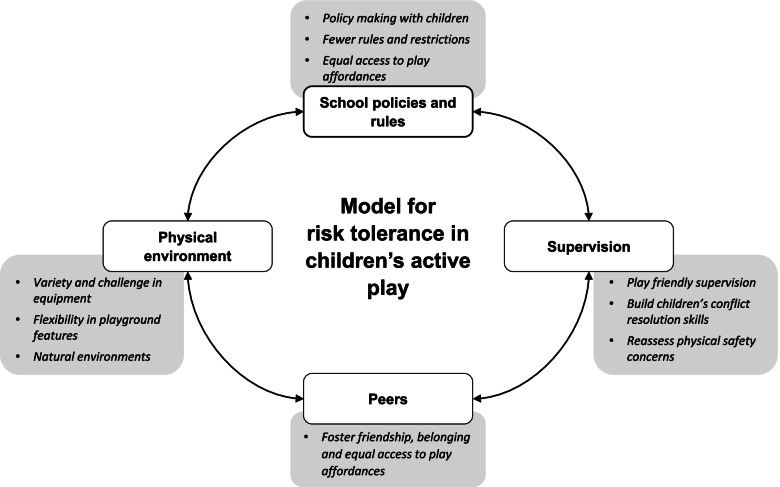
Model for risk tolerance in children’s active play

Our findings indicate several ways risk tolerance may support active play and improve children’s experience of recess. This includes greater autonomy for children in play, promoting play friendly supervision, social wellbeing, and equal access to play affordances, and providing more stimulating play environments, that include opportunities for risk-taking and challenge, flexibility in play, and nature. A model for risk tolerance in children’s active play is proposed in Fig. 4 to guide efforts in schools to optimise children’s play opportunities during recess.

### Recommendations for policy, practice, and future research

We present below several recommendations for policy and practice and important directions for future research.

1. **Foster a culture of risk tolerance in schools**, as proposed in Fig. 4, through playground interventions that target school policies and rules, the physical environment, supervision practices, and peer interaction.
2. **Consult and include children in decision-making that impacts their play environment:** Children have many constructive and innovative suggestions for promoting active play and a positive recess experience. Moreover, their perspectives often differ to adults, with adults failing to ‘see’ children’s places for play. Taking time to find out from children what happens in the playground before making changes is recommended [99, 126]. To this end, child-centred participatory research methods may provide a useful means for generating potential solutions to playground problems [33, 126, 136–138].
3. **Renegotiate playground rules:** Schools should consider where the boundary lies between necessary and unnecessary rules as children perceived adult authority limited their ability to implement their own ideas about how to play and be active. There will be cultural and contextual variation across schools, therefore, the combination of possibilities and constraints in relation to play will differ accordingly. As such, community-level and participatory-based approaches are recommended [33, 58, 126].
4. **Improve understanding of the relationship between children’s physical literacy levels and their ability to negotiate risk and safety in play.** Children’s ability to manage physical risk and keep themselves safe are important skills that cannot be acquired without an opportunity to practice and develop [51], however, only one study in this review specifically examined children’s risk-taking in play [101], and there is a lack of research examining the relationship between physical literacy and risky play in school age children.
5. **Investigate a wider range of ways unequal access to play affordances occurs in schools e.g., based on ethnicity/race or SES,** than was examined in included studies. Future enquiry should seek to address gaps in the literature around disparities and social determinants of play.

### Strengths and limitations

Major strengths of this review include the use of theory to guide the conceptual framework development and adherence to best-practice principles for the framework synthesis method [73]. Harnessing children’s perspectives and experiences through this synthesis is a strength and provides a knowledge base to support practitioners and policy makers. However, the search strategy did not include grey literature or studies that were published in languages other than English, therefore it is possible valuable information was overlooked. There were also limitations in the evidence, notably, many studies did not provide sufficient contextual and demographic information to make between study comparisons. While some studies included schools with a mix of SES profiles, they did not report findings by SES, making it difficult to clarify the role SES had in children’s access to play affordances. Similarly, although nine studies were conducted in both rural and urban settings, findings were not distinguished by urban/rural characteristics, and an additional eight studies did not describe the school setting by geographical location. Given that the review is qualitative and concentrates on children’s perceptions, primary studies did not seek to verify the play constraints children described. Indeed, to do so may run the risk of privileging adult perspectives over children’s (and in fact several studies identified playground rules were not explicitly written down in policy, and application varied between supervisors). Many studies did not report the researcher’s positionality, or the potential for influence during the research process. This is important for research with children, where a power imbalance exists and the potential for researchers to be leading in their questioning, for example, may be significant [139, 140]. Additionally, almost all studies were conducted in high income countries, and there is a need to understand more about active school play in lower- and middle-income countries. Finally, many of the studies were small which may limit generalisability of findings; however, this is compensated for to some degree by the number of studies included in the current review and diversity of settings and disciplines.

## Conclusions

This systematic review provides novel insights about the role of safety and risk in children’s active play during recess in schools, from the perspective of children themselves. The findings show a disparity between the play children wanted in schools and what they were able to do. Children enjoyed risk-taking and challenge in play and desired more freedom and a wider range of play affordances. However, they perceived socio-cultural factors (such as supervision practices, playground rules) constrained active play during recess, which were driven by adults’ concern with physical safety. These factors contributed to a cycle of risk-averse decision making and diminished affordances for play, which could have the inadvertent effect of exacerbating safety issues in the playground. A model for risk tolerance in active play was developed from the findings. Future work should balance the concerns of adults against the active play children want, involve children in decisions about playground policy, and foster a risk-tolerant culture in schools. In addition, the role of children’s physical literacy levels and their ability to negotiate risk and safety in play should be explored.

## Supplementary Information

**Additionalfile 1.** PRISMA Statement & ENTREQ Checklist**. **The completed statement and checklist.

**Additionalfile 2.** Search strategy**.** The search strategy, key concepts and search terms, and example database search.

**Additionalfile 3. **Initial conceptual framework and codebook**.** An explanation of the development process for the initial conceptual framework, together with thec odebook that guided the evidence synthesis.

**Additionalfile 4. **Characteristics of included studies**.** Table showing the characteristicsof included studies, including: Author, year, country, discipline, research aim, study design, theoretical framework, sampling methods, setting and participant characteristics, data collection and analysis methods, rigour.

**Additionalfile 5. **Quality appraisal of included studies**.** Table showing appraisal results for all studies using the CASP checklist.

## Data Availability

All data generated or analysed during this study are included in this published article and its supplementary information files.

## Abbreviations

ENTREQ: Enhancing Transparency in Reporting the Synthesis of Qualitative Research statement
CASP: Critical Appraisal Skills Programme Qualitative Checklist
CRC: United Nations Convention on the Rights of the Child
PA: Physical activity
PRISMA: Preferred Reporting Items for Systematic Reviews and Meta-Analyses checklist
SEM: Socio-ecological model

## References

[1] Dale LP (2019). Physical activity and depression, anxiety, and self-esteem in children and youth: An umbrella systematic review. Ment Health Phys Act.

[2] Poitras VJ (2016). Systematic review of the relationships between objectively measured physical activity and health indicators in school-aged children and youth. Appl Physiol Nutr Metab.

[3] Okely AD (2012). A systematic review to update the Australian physical activity guidelines for children and young people.

[4] Donnelly JE (2016). Physical activity, fitness, cognitive function, and academic achievement in children: a systematic review. Med Sci Sports Exerc.

[5] Singh AS (2019). Effects of physical activity interventions on cognitive and academic performance in children and adolescents: a novel combination of a systematic review and recommendations from an expert panel. Br J Sports Med.

[6] Sullivan RA (2017). The Association of Physical Activity and Academic Behavior: A Systematic Review. J Sch Health.

[7] Guthold R (2018). Worldwide trends in insufficient physical activity from 2001 to 2016: a pooled analysis of 358 population-based surveys with 1· 9 million participants. Lancet Glob Health.

[8] World Health Organization (2018). Global action plan on physical activity 2018–2030: more active people for a healthier world.

[9] Aubert S (2018). Global matrix 3.0 physical activity report card grades for children and youth: results and analysis from 49 countries. J Phys Act Health.

[10] Guthold R (2020). Global trends in insufficient physical activity among adolescents: a pooled analysis of 298 population-based surveys with 1·6 million participants. Lancet Child Adolescent Health.

[11] Yogman M (2018). The Power of Play: A Pediatric Role in Enhancing Development in Young Children. Pediatrics.

[12] UN Committee on the Rights of the Child. General comment No. 17 (2013) on the right of the child to rest, leisure, play, recreational activities, cultural life and the arts (art. 31). CRC/C/GC/17. 2013. Available from: https://www.refworld.org/docid/51ef9bcc4.html.29 June 2020.

[13] Whitebread D (2012). The Importance of Play.

[14] Pesce C (2016). Deliberate play and preparation jointly benefit motor and cognitive development: Mediated and moderated effects. Front Psychol.

[15] United Nations. Convention on the Rights of the Child. 1989. Available from: https://treaties.un.org/Pages/ViewDetails.aspx?src=IND&mtdsg_no=IV-11&chapter=4&lang=en. 7 July 2020.

[16] Janssen I (2014). Active play: An important physical activity strategy in the fight against childhood obesity. Can J Public Health.

[17] Truelove S, Vanderloo LM, Tucker P (2017). Defining and measuring active play among young children: A systematic review. J Phys Act Health.

[18] Pedroni C (2019). Environmental correlates of physical activity among children 10 to 13 years old in Wallonia (Belgium). BMC Public Health.

[19] Aubert S (2018). Report Card grades on the physical activity of children and youth comparing 30 very high Human Development Index countries. J Phys Act Health.

[20] Hyndman B (2017). Contemporary School Playground Strategies for Healthy Students.

[21] Ramstetter CL, Murray R, Garner AS (2010). The crucial role of recess in schools. J Sch Health.

[22] Chancellor B (2013). Primary school playgrounds: features and management in Victoria Australia. Int J Play.

[23] Mullan K (2019). A child's day: trends in time use in the UK from 1975 to 2015. Br J Sociol.

[24] Barros RM, Silver EJ, Stein RE (2009). School recess and group classroom behavior. Pediatrics.

[25] UN Treaty Body Database. Ratification Status for CRC - Convention on the Rights of the Child. 2021. Available from: https://tbinternet.ohchr.org/_layouts/15/TreatyBodyExternal/Treaty.aspx?Treaty=CRC&Lang=en. 22 Spetmber 2021.

[26] CDC and Springboard to Active Schools. Data Brief: Keep Recess in Schools. 2019; Available from: https://www.cdc.gov/healthyschools/physicalactivity/pdf/Recess_Data_Brief_CDC_Logo_FINAL_191106.pdf.

[27] Baines E, Blatchford P (2019). School break and lunch times and young people’s social lives: A follow-up national study.

[28] McNamara L, PHE Canada (2020). The Role of Recess in Canadian Elementary Schools: A National Position Paper.

[29] Australian Government. Australian Education Act 2013. Complilation No.7. 2020. Available from: https://www.legislation.gov.au/Details/C2020C00142. 22 September 2021.

[30] Ramstetter, C., et al. Global Recess Alliance: Statement on Recess. 2020. Available from: https://globalrecessalliance.org/recess-statement/. 22 September 2021.

[31] Messing S (2019). How can physical activity be promoted among children and adolescents? A systematic review of reviews across settings. Frontiers in Public Health.

[32] Stanley RM, Boshoff K, Dollman J (2012). Voices in the playground: A qualitative exploration of the barriers and facilitators of lunchtime play. J Sci Med Sport.

[33] Caro HEE (2016). Dutch Primary Schoolchildren's Perspectives of Activity-Friendly School Playgrounds: A Participatory Study. Int J Environ Res Public Health.

[34] Parrish AM (2020). Interventions to Change School Recess Activity Levels in Children and Adolescents: A Systematic Review and Meta-Analysis. Sports Med.

[35] Massey W, Neilson L, Salas J (2019). A critical examination of school-based recess: what do the children think?. Qualitative Res Sport Exerc Health.

[36] Lee E-Y (2021). Systematic review of the correlates of outdoor play and time among children aged 3–12 years. Int J Behav Nutr Phys Act.

[37] Herrington S, Brussoni M (2015). Beyond Physical Activity: The Importance of Play and Nature-Based Play Spaces for Children's Health and Development. Curr Obes Rep.

[38] Brussoni M (2015). What is the relationship between risky outdoor play and health in children? A systematic review. Int J Environmental Res Public Health.

[39] Sandseter EBH, Kennair LEO (2011). Children's risky play from an evolutionary perspective: The anti-phobic effects of thrilling experiences. Evol Psychol.

[40] Little H, Wyver S (2008). Outdoor play: Does avoiding the risks reduce the benefits?. Australas J Early Childhood.

[41] Wyver S (2010). Ten ways to restrict children's freedom to play: The problem of surplus safety. Contemp Issues Early Child.

[42] Tremblay M (2015). Position statement on active outdoor play. Int J Environ Res Public Health.

[43] Beck U (1992). Risk Society: toward a New Modernity.

[44] Ball DJ (2019). Avoiding a dystopian future for children's play. International Journal of Play.

[45] Furedi F (2002). Culture of Fear: Risk taking and the morality of low expectation.

[46] Gill T (2007). No Fear: Growing up in a risk averse society.

[47] Malone K (2007). The bubble-wrap generation: children growing up in walled gardens. Environ Educ Res.

[48] Pynn SR (2019). An intergenerational qualitative study of the good parenting ideal and active free play during middle childhood. Children's Geographies.

[49] Clements R (2004). An Investigation of the Status of Outdoor Play. Contemp Issues Early Child.

[50] Little H (2017). Promoting risk-taking and physically challenging play in Australian early childhood settings in a changing regulatory environment. Journal of Early Childhood Research.

[51] Brussoni M (2012). Risky Play and Children's Safety: Balancing Priorities for Optimal Child Development. Int J Environ Res Public Health.

[52] Woolley H, Lowe A (2013). Exploring the Relationship between Design Approach and Play Value of Outdoor Play Spaces. Landsc Res.

[53] Sandseter EBH (2010). *Scaryfunny: A Qualitative Study of Risky Play Among Preschool Children*, in *Department of Psychology*.

[54] Sandseter EBH (2020). Barriers for Outdoor Play in Early Childhood Education and Care (ECEC) Institutions: Perception of Risk in Children’s Play among European Parents and ECEC Practitioners. Child Care Pract.

[55] Stephenson A (2003). Physical risk-taking: dangerous or endangered?. Early Years.

[56] Bundy A (2017). Sydney Playground Project: A Cluster-Randomized Trial to Increase Physical Activity, Play, and Social Skills. J Sch Health.

[57] Hesketh KR, Lakshman R, van Sluijs E (2017). Barriers and facilitators to young children's physical activity and sedentary behaviour: a systematic review and synthesis of qualitative literature. Obes Rev.

[58] Lee H, et al. A meta-study of qualitative research examining determinants of children's independent active free play. International Journal of Behavioral Nutrition and Physical Activity. 2015;12(5):1-12. 10.1186/s12966-015-0165-9 PMC4318368 25616690

[59] Hyndman BP, Benson A, Telford A (2016). Active play: exploring the influences on children's school playground activities. Am J Play.

[60] Tuckerman J, Kaufman J, Danchin M (2020). How to use qualitative methods for health and health services research. J Paediatr Child Health.

[61] Hennink M, Hutter I, Bailey A (2020). Qualitative Research Methods.

[62] Page MJ (2021). The PRISMA 2020 statement: an updated guideline for reporting systematic reviews. BMJ.

[63] Tong A (2012). Enhancing transparency in reporting the synthesis of qualitative research: ENTREQ. BMC Med Res Methodol.

[64] Sandseter EBH (2007). Categorising risky play—how can we identify risk-taking in children's play?. Eur Early Child Educ Res J.

[65] Evans J (2003). Changes to (primary) school recess and their effect on children's physical activity: an Australian perspective. J Phys Educ N Z.

[66] Evans J (2007). Whatever happened to playtime? [Are organised games during recesses and lunch breaks necessary?]. Educ Res Perspectives.

[67] Veritas Health Innovation, Covidence systematic review sofware. Melbourne: Covidence; 2019.

[68] Ridgers ND, Stratton G, Fairclough SJ (2006). Physical activity levels of children during school playtime. Sports Med.

[69] ISO 31000. Risk management — Guidelines. 2018. Available from: https://www.iso.org/obp/ui/#iso:std:iso:31000:ed-2:v1:en. 19 March 2021.

[70] Québec WHO Collaborating Centre for Safety Promotion and Injury Prevention. Safety and Safety Promotion: Conceptual and Operational Aspects. 1998. Available from https://www.inspq.qc.ca/pdf/publications/150_SecurityPromotion.pdf. 19 March 2021.

[71] Higgins JPT, Thomas J (2019). Cochrane Handbook for Systematic Reviews of Interventions.

[72] Brunton G, Oliver S, Thomas J (2020). Innovations in framework synthesis as a systematic review method. Res Synthesis Methods.

[73] Gough D, Oliver S, Thomas J (2017). An Introduction to Systematic Reviews.

[74] Bronfenbrenner U (1994). Ecological models of human development, in International Encyclopedia of Education.

[75] Gibson JJ (1979). The Ecological Approach to Visual Perception.

[76] Barnett-Page E, Thomas J (2009). Methods for the synthesis of qualitative research: a critical review. BMC Med Res Methodol.

[77] Boyle DE, Marshall NL, Robeson WW (2003). Gender at play: Fourth-grade girls and boys on the playground. Am Behav Sci.

[78] Critical Appraisal Skills Programme. CASP Qualitative Checklist. 2018. Available from: https://casp-uk.net/wp-content/uploads/2018/01/CASP-Qualitative-Checklist-2018.pdf.10 September 2020.

[79] Majid U, Vanstone M (2018). Appraising Qualitative Research for Evidence Syntheses: A Compendium of Quality Appraisal Tools. Qual Health Res.

[80] Long HA, French DP, Brooks JM (2020). Optimising the value of the critical appraisal skills programme (CASP) tool for quality appraisal in qualitative evidence synthesis. Res Methods Med Health Sci.

[81] Carroll C, Booth A (2015). Quality assessment of qualitative evidence for systematic review and synthesis: Is it meaningful, and if so, how should it be performed?. Res Synth Methods.

[82] Thomas J, Harden A (2008). Methods for the thematic synthesis of qualitative research in systematic reviews. BMC Med Res Methodol.

[83] Braun V, Clarke V (2019). Reflecting on reflexive thematic analysis. Qualitative Res Sport Exerc Health.

[84] Lupton D. Risk: Second Edition. Oxon: Routledge; 2013.

[85] McWhannell N, Triggs C, Moss S (2019). Perceptions and measurement of playtime physical activity in English primary school children: The influence of socioeconomic status. Eur Phys Educ Rev.

[86] Pearce G, Bailey RP (2011). Football pitches and Barbie dolls: young children’s perceptions of their school playground. Early Child Dev Care.

[87] Powell E, Woodfield LA, Nevill AAM (2016). Children’s physical activity levels during primary school break times: A quantitative and qualitative research design. Eur Phys Educ Rev.

[88] Thomson S (2005). ‘Territorialising’ the primary school playground: deconstructing the geography of playtime. Children's Geographies.

[89] Thomson S (2007). Do’s and don’ts: children’s experiences of the primary school playground. Environ Educ Res.

[90] Hemming P (2007). Renegotiating the Primary School: Children's Emotional Geographies of Sport. Exerci Active Play Children's Geographies.

[91] Jarvis P (2007). Dangerous Activities within an Invisible Playground: A Study of Emergent Male Football Play and Teachers' Perspectives of Outdoor Free Play in the Early Years of Primary School. Int J Early Years Educ.

[92] Thomson S (2003). A well-equipped hamster cage: The rationalisation of primary school playtime. Education 3-13.

[93] Hyndman B (2012). Moving Physical Activity Beyond the School Classroom: A Social-ecological Insight for Teachers of the facilitators and barriers to students' non-curricular physical activity. Australian J Teach Educ.

[94] Hyndman BP, Telford A (2015). Should Educators Be 'Wrapping School Playgrounds in Cotton Wool' to Encourage Physical Activity? Exploring Primary and Secondary Students' Voices from the School Playground. Australian J Teach Educ.

[95] Snow D (2019). Girls’ perspectives on the ideal school playground experience: an exploratory study of four Australian primary schools. Children's Geographies.

[96] Ndhlovu S, Varea V (2018). Primary School Playgrounds as Spaces of Inclusion/Exclusion in New South Wales Australia. Education 3-13.

[97] Parrish AM (2012). Using interviews and peer pairs to better understand how school environments affect young children's playground physical activity levels: a qualitative study. Health Educ Res.

[98] Willenberg LJ (2010). Increasing school playground physical activity: A mixed methods study combining environmental measures and children's perspectives. J Sci Med Sport.

[99] Pawlowski CS (2019). Changing recess geographies: children’s perceptions of a schoolyard renovation project promoting physical activity. Children's Geographies.

[100] Pawlowski CS (2019). Designing Activating Schoolyards: Seen from the Girls’ Viewpoint. Int J Environ Res Public Health.

[101] Christensen P, Mikkelsen MR (2008). Jumping off and being careful: Children's strategies of risk management in everyday life. Sociol Health Illn.

[102] Rasmussen K (2004). Places for Children - Children's Places. Childhood Global J Child Res.

[103] Harvey J (2018). Exploring the perspectives of 10-, 11-, and 12-year-old primary school students on physical activity engagement—“'Cause you can't just be sitting at a desk all the time!”. Child Care Health Develop.

[104] Button BL, Tillmann S, Gilliland J (2020). Exploring children's perceptions of barriers and facilitators to physical activity in rural Northwestern Ontario Canada. Rural Remote Health.

[105] McNamara L (2013). What's Getting in the Way of Play? An Analysis of the Contextual Factors that Hinder Recess in Elementary Schools. Canadian J Action Res.

[106] Ren JY, Langhout RD (2010). A recess evaluation with the players: taking steps toward participatory action research. Am J Community Psychol.

[107] Massey WV (2020). Observations from the playground: Common problems and potential solutions for school-based recess. Health Educ J.

[108] Sharkey JD (2014). Effective yard supervision: From needs assessment to customized training. Contemp Sch Psychol.

[109] Eskola S (2018). Children's perceptions of factors related to physical activity in schools. Educ Res.

[110] Norðdahl K, Einarsdóttir J (2015). Children's Views and Preferences Regarding Their Outdoor Environment. J Adventure Educ Outdoor Learning.

[111] Martínez-Andrés M (2017). “Football is a boys’ game”: children’s perceptions about barriers for physical activity during recess time. Int J Qual Stud Health Well Being.

[112] Gyllencreutz L (2020). Injury risks during outdoor play among Swedish schoolchildren: teachers’ perceptions and injury preventive practices. Education 3-13.

[113] Clements R, Messanga M, Millbank A (2008). Traditional Children's Games in Tanzania. Child Youth Environ.

[114] Heft H (1988). Affordances of children's environments: A functional approach to environmental description. Children's Environ Q.

[115] Sandseter EBH (2009). Affordances for risky play in preschool: The importance of features in the play environment. Early Childhood Educ J.

[116] Kleppe R, Melhuish E, Sandseter EBH (2017). Identifying and characterizing risky play in the age one-to-three years. Eur Early Child Educ Res J.

[117] Jelleyman C (2019). A cross-sectional description of parental perceptions and practices related to risky play and independent mobility in children: The New Zealand state of play survey. Int J Environ Res Public Health.

[118] Sport Australia, Australian Physical Literacy Framework. Canberra: Australian Sports Commission; 2019.

[119] Rutter H (2017). The need for a complex systems model of evidence for public health. Lancet.

[120] Sandseter EBH, Kleppe R, Sando OJ. The Prevalence of Risky Play in Young Children’s Indoor and Outdoor Free Play. Early Childhood Educ J. 2021;49:303-312.

[121] Kyttä M (2004). The extent of children's independent mobility and the number of actualized affordances as criteria for child-friendly environments. J Environ Psychol.

[122] Australian Sports Commission. Physical Literacy Definition. 2017. Available from: https://www.clearinghouseforsport.gov.au/__data/assets/pdf_file/0008/774026/ASC_34651_Physical_Literacy_Consensus_Statement_FA2.pdf.Accessed 29 Sep 2019.

[123] Bellew, B., C. Rose, and L Reece, Active and Inactive Young Australians. An Independent Review of Research into Enablers and Barriers to Participation in Sport, Active Recreation and Physical Activity among Children and Adolescents (2020). Produced for the NSW Office of Sport by the SPRINTER Research Group.

[124] Dodd HF, Lester KJ. Adventurous play as a mechanism for reducing risk for childhood anxiety: A conceptual model. Clin Child Family Psychol Rev. 2021;24(1):164-181. 10.1007/s10567-020-00338-w PMC7880968 33464448

[125] Fjortoft I (2004). Landscape as Playscape: The Effects of Natural Environments on Children's Play and Motor Development. Child Youth Environ.

[126] Ardelean AK, Smith K, Russell W (2021). The Case for Play in Schools: A review of the literature.

[127] Rönnlund M (2015). Schoolyard stories: Processes of gender identity in a ‘children’s place’. Childhood Global J Child Res.

[128] Spark C, Porter L, de Kleyn L (2019). ‘We’re not very good at soccer’: gender, space and competence in a Victorian primary school. Children's Geographies.

[129] Aminpour F, Bishop K, Corkery L. The hidden value of in-between spaces for children’s self-directed play within outdoor school environments. Landscape and Urban Planning. 2020;194:1-16.

[130] Niehues AN (2013). Everyday uncertainties: reframing perceptions of risk in outdoor free play. J Adventure Educ Outdoor Learning.

[131] Little H, Sandseter EBH, Wyver S (2012). Early childhood teachers' beliefs about children's risky play in australia and norway. Contemp Issues Early Child.

[132] Bundy AC (2009). The risk is that there is ‘no risk’: a simple, innovative intervention to increase children’s activity levels. Int J Early Years Educ.

[133] Hills AP, Dengel DR, Lubans DR (2015). Supporting Public Health Priorities: Recommendations for Physical Education and Physical Activity Promotion in Schools. Prog Cardiovasc Dis.

[134] Sandseter EBH, Little H, Wyver S (2012). Do theory and pedagogy have an impact on provisions for outdoor learning? A comparison of approaches in Australia and Norway. J Adventure Educ Outdoor Learning.

[135] Sandseter EBH, Sando OJ (2016). "We Don't Allow Children to Climb Trees" How a Focus on Safety Affects Norwegian Children's Play in Early-Childhood Education and Care Settings. Am J Play.

[136] Bristow S, Atkinson C (2020). Child-led research investigating social, emotional and mental health and wellbeing aspects of playtime. Educ Child Psychol.

[137] Tyrie J (2019). Power, rights and play: control of play in school grounds, an action research project from Wales. Education 3-13.

[138] Hayball FZL, Pawlowski CS (2018). Using participatory approaches with children to better understand their physical activity behaviour. Health Educ J.

[139] Connelly P. Race, gender and critical reflexivity in research with young children, in Research with Children: Perspectives and Practices (3rd ed.), P. Christensen and A. James, Editors. Oxon: Routledge; 2017.

[140] Christensen PH (2004). Children's participation in ethnographic research: Issues of power and representation. Child Soc.

