# Supplementary material for: “All the fun stuff, the teachers say, ‘that’s dangerous!’” Hearing from children on safety and risk in active play in schools: a systematic review
Source: Int J Behav Nutr Phys Act. 2022 Jun 25;19:72. doi: 10.1186/s12966-022-01305-0 (PMC9233824; doi:10.1186/s12966-022-01305-0)
Supplement: Supplementary file 2 — Additionalfile 2. Search strategy. The search strategy, key concepts and search terms, and example database search. [file 12966_2022_1305_MOESM2_ESM.docx]

# Additional file 2: Search strategy

The search strategy was developed with the assistance of a university health librarian with expertise in advanced database searching and aimed to locate published literature only. An initial limited search of ERIC, MEDLINE Complete, APA PsycInfo and SPORTDiscus with Full-text was undertaken to identify articles on the topic. The text words contained in the titles and abstracts of relevant articles, and the index terms used to describe the articles were used to develop a full search strategy for six bibliographic databases: Education Source, ERIC, MEDLINE Complete, APA PsycInfo, SPORTDiscus with Full-text, and Embase. The search strategy, including all identified keywords and relevant Emtree, MeSH and Thesaurus terms, was adapted for each included information source. An example is given below for MEDLINE Complete via the EBSCOhost database. The final search was completed on 26/03/21.

Restrictions applied:

- Language: English language only
- Publication or release period: 2000 onwards - Studies published from 2000 to the present will be included to capture contemporary views and observations of children’s active play and/or risky play in schools since changing attitudes to safety and risk in children’s play have been documented [1-3].”
- The term ‘risky play’ is a relatively recent term, first defined in 2007 [4] to describe types of play that were once a regular feature in children’s play but have become less common in recent generations, primarily due to changes in societal attitudes to safety and risk in childhood.

**Key concepts and search terms**

|  | **Term** | **Alternative terms** |
| --- | --- | --- |
| **Concept 1** | Child | Student, pupil, youth, adolescent |
| Additional terms within this concept | Teacher | Supervisor, principal |
| Additional terms within this concept | Parent |  |
| **Concept 2** | School |  |
| **Concept 3** | Active play | Outdoor play, free play, unstructured play, child play, risky play, challenging play, adventurous play |
| Additional terms within this concept | Recess | Breaktime, break time, playtime, play time, lunchtime, lunch time |
| Additional terms within this concept | Playground | School ground, schoolyard, school yard |

**Subject headings**

|  | **Medline (MeSH)** | **ERIC**  **(Thesaurus)** | **Psych Info**  **(Thesaurus)** | **Sport-discus**  **(Thesaurus)** | **Embase (Emtree)** | **Education Source**  **(Thesaurus)** |
| --- | --- | --- | --- | --- | --- | --- |
| **Concept 1:**  **Child** | Child,  Adolescent,  Students,  School Teachers,  Parents | Student attitude,  Teacher attitudes,  Principals,  Parent attitudes | Student attitudes,  Teacher attitudes,  Teacher student interaction,  Parental attitudes | School children,  Teachers | School child,  School teacher,  Parent | Childhood attitudes,  Teacher attitudes,  Elementary school principals,  Middle school principals,  Parent attitudes |
| **Concept 2:**  **School** | Schools | Elementary schools,  Middle schools | Elementary schools,  Middle schools | Schools | Schools | Elementary schools,  Middle schools |
| **Concept 3:**  **Active play** | Play and Playthings | Play,  Playgrounds,  Recess breaks | Playgrounds | School recess breaks,  Playgrounds | Play,  Playground | Play,  Play environment,  Playground equipment,  School recess breaks,  School grounds |

**Example electronic Database search: MEDLINE Complete via EBSCOhost**

| Concept 1 – field labels | S1 | TI child* OR AB child* |
| --- | --- | --- |
|  | S2 | TI youth* OR AB youth* |
|  | S3 | TI adolescen* OR AB adolescen* |
|  | S4 | TI pupil* OR AB pupil* |
|  | S5 | TI student* OR AB student* |
| Additional terms within concept 1 | S6 | TI teacher* OR AB teacher* |
|  | S7 | TI supervis* OR AB supervis* |
|  | S8 | TI principal* OR AB principal* |
|  | S9 | TI parent* OR AB parent* |
| Concept 1 – Subject labels (MeSH) | S10 | MH “Child” |
|  | S11 | MH “adolescent” |
|  | S12 | MH “Students” |
|  | S13 | MH “School Teachers” |
|  | S14 | MH “Parents+” |
| Combine terms with ‘OR’ | S15 | S1 OR S2 OR S3 OR S4 OR S5 OR S6 OR S7 OR S8 OR S9 OR S10 OR S11 OR S12 OR S13 OR S14 |
| Concept 2 – field labels | S16 | TI school* OR AB school* |
| Concept 2 – Subject labels (MeSH) | S17 | MH “schools” |
| Combine terms with ‘OR’ | S18 | S16 OR S17 |
| Concept 3 – field labels | S19 | TI “active play*” OR AB “active play*” |
|  | S20 | TI “outdoor play*” OR AB “outdoor play*” |
|  | S21 | TI “free play*” OR AB “free play*” |
|  | S22 | TI “unstructured play*” OR AB “unstructured play*” |
|  | S23 | TI “child* play*” OR AB “child* play*” |
|  | S24 | TI “risky play*” OR AB “risky play*” |
|  | S25 | TI “adventur* play*” OR AB “adventur* play*” |
|  | S26 | TI “challenging play*” OR AB “challenging play*” |
| Additional terms within concept 3 | S27 | TI Recess OR AB Recess |
|  | S28 | TI Breaktime* OR AB Breaktime* |
|  | S29 | TI “Break time*” OR AB “Break time*” |
|  | S30 | TI Playtime* OR AB Playtime* |
|  | S31 | TI “Play time*” OR AB “Play time*” |
|  | S32 | TI Lunchtime* OR AB Lunchtime* |
|  | S33 | TI “Lunch time*” OR AB “Lunch time*” |
|  | S34 | TI playground* OR AB playground* |
|  | S35 | TI “play ground*” OR AB “play ground*” |
|  | S36 | TI schoolyard* OR AB schoolyard* |
|  | S37 | TI “school yard*” OR AB “school yard*” |
|  | S38 | TI “school ground*” OR AB “school ground*” |
| Concept 3 – Subject labels (MeSH) | S39 | MH “play and playthings+” |
| Combine terms with ‘OR’ | S40 | S19 OR S20 OR S21 OR S22 OR S23 OR S24 OR S25 OR S26 OR S27 OR S28 OR S29 OR S30 OR S31 OR S32 OR S33 OR S34 OR S35 OR S36 OR S37 OR S38 OR S39 |
| Combine terms with ‘AND’ | S41 | S15 AND S18 AND S40 |
|  |  |  |

References

1. Stephenson, A., *Physical risk-taking: dangerous or endangered?* Early Years, 2003. **23**(1): p. 35-43.

2. Evans, J., *Changes to (primary) school recess and their effect on children's physical activity: an Australian perspective.* Journal of Physical Education New Zealand, 2003. **36**(1): p. 53-62.

3. Evans, J., *Whatever happened to playtime? [Are organised games during recesses and lunch breaks necessary?].* Education Research and Perspectives, 2007. **34**(1): p. 198-211.

4. Sandseter, E.B.H., *Categorising risky play—how can we identify risk‐taking in children's play?* European Early Childhood Education Research Journal, 2007. **15**(2): p. 237-252.
