# Supplementary material for: “All the fun stuff, the teachers say, ‘that’s dangerous!’” Hearing from children on safety and risk in active play in schools: a systematic review
Source: Int J Behav Nutr Phys Act. 2022 Jun 25;19:72. doi: 10.1186/s12966-022-01305-0 (PMC9233824; doi:10.1186/s12966-022-01305-0)
Supplement: Supplementary file 4 — Additionalfile 4. Characteristics of included studies. Table showing the characteristicsof included studies, including: Author, year, country, discipline, research aim, study design, theoretical framework, sampling methods, setting and participant characteristics, data collection and analysis methods, rigour. [file 12966_2022_1305_MOESM4_ESM.docx]

# Additional file 4: Characteristics of included studies

| **Author, Year,**  **Country** | **Discipline** | **Research Aims** | **Study Design & Theoretical Framework** | **Sampling Methods:**  **School Setting & Participants** | **School Setting Characteristics** (n=sample size) | **Participant Characteristics** (n=sample size) | **Qualitative Data Collection** | **Qualitative data analysis** | **Rigour** |
| --- | --- | --- | --- | --- | --- | --- | --- | --- | --- |
| Button et al. 2020 [1],  **Canada** | Public health: PA promotion | To explore rural children’s perspectives on barriers and facilitators to PA. | Child-centred approach,  SEM | Schools: Recruited as part of STEAM Project.  Participants: Self-selected after researcher gave project presentation to Grades 4-8. | Elementary (n=**4**),  rural, NW Ontario,  43% Indigenous students. | Students (n=**84),**  8-14 years,  51% female,  51% white | FG, semi-structured  Field notes | Thematic analysis, using Braun & Clarke’s 6-step process | Critical friend, reflexive practice |
| Caro et al. 2016 [2],  **Netherlands** | Public health: PA promotion | To explore child-identified determinants of activity-friendly school playgrounds | Participatory research design | Schools: Screened for openness to improve playgrounds.  Participants: Random sampling of students that self-selected | Elementary (n=**3**), urban, Amsterdam,  1 each Low, Med, High SES. | Students (n=**18**),  9-12 years,  56% female | Child-led methods: Group meetings, Photos, Drawings, Field notes, Children's journal with questionnaire | Iterative inductive and deductive coding, themes developed | Member checking, method triangulation, collaborative review of themes |
| Christiansen et al. 2008 [3],  **Denmark** | Child development and play | To explore the individual and collective agency of children when encountering chance and risk in everyday life. | Ethnography, Sociological theories of risk | Schools: NR  Participants: Purposive based on inclusion criteria (criteria not published) | Elementary (n=**1**), urban, Copenhagen. | Students (n=**35)**,  10-12 years | Observation and Field notes, FG,  Informal conversations,  Guided tours | Ethnographic analysis | Eight-month immersion in research context |
| Clements et al. 2008 [4],  **Tanzania** | Child development and play | To describe traditional play activities of primary school-age children in Tanzania, East Africa. | NR | NR | Elementary (n=**1**), urban, Mwanza city. | Students (n=NR),  4-12 years | Ethnographic Observation,  Field testing,  Questionnaires | NR | NR |
| Eskola et al. 2018 [5],  **Finland** | Public health: PA promotion | To investigate the factors perceived by children from their school environment that promote or inhibit PA during their school day. | Case study, EnRG model | Schools: Recruited as part of larger HEPCOM project:  Participants: Purposively selected 1 class from the pilot HEPCOM school | Elementary (n=**1**), Central Finland. | Students (n=**22**),  8-9 years,  64% female,  100% Finnish born | FG,  Photo-elicitation (children’s playground photos) | Inductive content analysis | Pilot interviews, collaborative review of themes |
| Gyllencreutz et al. 2020 [6],  **Sweden ^ⴕ^** | Injury prevention | To explore injury risk situations among school children during  outdoor play in the school environment | Field study design,  Theory of social representations | Schools: Purposive based on playground type.  Participants: Convenience sampling, all children and teachers from relevant grades invited to participate. | Elementary (n=**2**), urban, Sub-artic Northern Sweden,  Small forest, ice skating rinks, tree houses in schoolyard. | Students (n=**46**),  6-7 and 11-12 years | Naturalistic observations,  FG,  Field notes | Content analysis, using an observation scheme and play coding structure | Data source triangulation, method triangulation |
| Harvey et al. 2018 [7],  **Canada** | Public health: PA promotion | To explore the perspectives of primary students regarding the facilitators, barriers, and recommendations for PA engagement at their schools. | Qualitative methodology | Schools: Purposefully selected through local health unit, school board.  Participants: self-selected after classroom visits by researchers. | Elementary, rural (n=**3**) and urban (remote Canadian city; n=**3**). | Students (n=**53**),  10-12 years,  45% female,  25% Aboriginal | FG, semi structured  Field notes | Inductive content analysis | Investigator triangulation, member reflections, note taking, independent data  analysis |
| Hemming et al. 2007 [8],  **England** | Public health: PA promotion | To explore the issue of sport, exercise, and active play in the primary school, in the context of the UK national policy concern for health. | Instrumental case study approach, Child-centred participatory methods | Schools: Purposive based on varied physical contexts for sport and active play.  Participants: NR | Elementary (n=**1**), urban, Midlands region. | Students (n=**23**),  9-10 years,  male and female (% NR) | Observation,  Paired interviews, semi-structured,  Individual drawings, Group photography | Data analysed using thematic categories & ethnographic data | Method triangulation, research diary |
| Hyndman et al. 2012 [9],  **Australia** | Public health: PA promotion | To explore the influences on and preferences of school students’ PA behaviour beyond the classroom. | Case study design,  SEM | Schools: Randomly selected from defined geographical area, 1 school purposively selected.  Participants: Self-selected from grades invited to participate. | Elementary (n=**2**),  Secondary (n=**2**),  Western Victoria,  1 Low, 1 Med SES from each school type. | Students (n=**78**),  10-13 years,  50% female | FG, semi-structured,  Map drawing | Data coded and categorized deductively according to SEM. | Method triangulation |
| Hyndman et al. 2015 [10],  **Australia** | Public health: PA promotion | To explore school students’ perceptions of the playground safety influences on PA participation during school breaks. | SEM | Schools: Randomly selected from defined geographical area, 1 school purposively selected.  Participants: Self-selected from grades invited to participate. | Elementary (n=**2**),  Secondary (n=**2**),  Western Victoria,  1 Low, 1 Med SES from each school type. | Elementary Students (n=**32**),  10-11 years, 50% female  Secondary students (n=**22**), 12-13 years, 50% female | FG, semi-structured | Data coded and categorized deductively according to SEM. | NR |
| Jarvis et al. 2007 [11],  **England ^ⴕ^** | Child development and play | To investigate the narratives underpinning rough and tumble play in the early years of primary school. | Ethnography,  Bio-culturalism | Schools: NR  Participants: NR | Elementary (n=**1**),  Northern England. | Students (n=**18**),  4-6 years,  50% female | Observation,  Field notes,  Informal conversations | NR | 18-month immersion in the research context |
| Martinez et al. 2017 [12],  **Spain** | Public health: PA promotion | To find out the factors that influence boys’ and girls’ perceptions for performing PA during playground recess. | SEM | Schools: Recruited as part of larger MOVI-2 project.  Participants: Purposive based on inclusion criteria (mix of gender, SES, rural and urban). | Elementary (n=**20**),  urban & rural Cuenca,  Low, Med & High SES. | Students (n=**98**),  8-11 years,  63% female | FG  Map drawings | Content analysis, continuous comparative method | Pilot FG, Investigator triangulation, negative case analysis |
| Massey et al. 2020 [13],  **USA** | Child development and play | To examine common problems observed during recess and translate into evidence-based, practical solutions for schools. | Observational study | Schools: Recruited as part of a larger project (unnamed).  Participants: NR | Elementary (n=**25**),  urban, rural, metro (Midwest, Northwest, Southwest),  Low, Med & High SES. | Students (n=**NR**),  5-11 years | Observation (GRF-OT Tool),  Field notes,  Playground drawings | Inductive content analysis | Critical reflections, iterative  consensus process, collaborative review of themes |
| McNamara et al. 2013 [14],  **Canada ^ⴕ^** | Child development and play | To highlight children’s voices about their recess environment, and add perspectives from the teachers, principals, and playground volunteers. | Inductive qualitative approach | Schools: Purposive sampling based on risk factors for low education outcomes.  Participants: Purposive (maximum variation sampling) to gain a range of perspectives. | Elementary (n=**4**),  South Ontario,  Low SES. | Students (n=**103**),  8-13 years | Questionnaires, with open-ended questions,  Observations,  Field notes,  Journals,  Interviews, unstructured | Data coded inductively; themes developed | Member checking, data source triangulation, method triangulation |
| McWhannel et al. 2019 [15],  **England** | Public health: PA promotion | To assess and compare playtime PA levels and explore perceptions of PA from two schools of different socioeconomic status. | Explorative study design, Mixed methods | Schools: Purposive sampling based on SES indicators.  Participants: NR | Elementary (n=**2**),  urban,  1 Low, 1 High SES. | Students (n=**32**),  7-8 years,  63% female | FG, semi-structured | Thematic analysis, using theoretical, deductive coding | Critical friends, reflexive practice |
| Ndhlovu et al. 2018 [16],  **Australia** | Child development and play | To determine how the design and organisation of primary school playground spaces may result in inclusion or exclusion of some groups of children | NR | Schools: Purposive based on school population (Indigenous, CALD).  Participants: NR | Elementary (n=**2**),  rural NSW,  High Indigenous and CALD populations. | Students (n=**30**),  5-12 years | Observation,  Field notes  Interviews, unstructured & interactive | Content analysis, using inductive coding. | Data source triangulation, collaborative review of themes, negative case analysis |
| Norodahl et al. 2015 [17],  **Iceland ^ⴕ^** | Environmental education and health | To explore children’s preferences about outdoor activities and surroundings in the outdoor school environment. | Participatory research design to inform school grounds project,  Theory of affordances | Schools: Recruited as part of a larger project on Education for sustainable development.  Participants: students selected by teachers using maximum variation approach (gender, age, neighborhoods, interests). | Elementary (n=**1**),  and preschool (n=**2**), urban, small municipality near Reykjavík. | Elementary Students (n=**8**),  7-9 years, 50% female.  Preschool students (n=**8**), 4-5 years,  50% female | Interviews, semi-structured, paired,  Walking tours,  Observations,  Field notes | Thematic analysis, using Braun & Clarke’s 6-step process | Data source triangulation, method triangulation |
| Parrish et al. 2012 [18],  **Australia ^ⴕ^** | Public health: PA promotion | To understand how physical and psychosocial school environmental variables influence children’s playground PA levels. | Qualitative descriptive design,  SCT | Schools: Extreme case sampling method (least and highest active schools in larger study).  Participants: Children from Grades 4-6 self-selected; One administrator from each school; Teachers NR. | Elementary (n=**6**),  Low & Med SES, High and low PA schools. | Students (n=**50**),  9-12 years,  58% female | Interviews, semi-structured, paired | Data coded inductively; themes developed. | Data source triangulation, method triangulation (from the larger study). |
| Pawlowski et al. 2019a [19],  **Denmark** | Public health: PA promotion | To investigate children’s perceptions of their new schoolyards, providing qualitative evidence from a post-intervention evaluation of a schoolyard renewal project. | Process evaluation of quasi-experimental intervention,  Affordance theory, Concept of place | Schools: Recruited as part of larger project, schools selected from across Denmark.  Participants: Random sampling of children whose parents had consented at intervention schools. | Elementary & Lower Secondary (n=**5**), urban & rural,  High & Low SES, Small & large student population & M^2^. | Students (n=**57**),  10-15 years,  49% female | FG, unstructured,  Walking tour of playground | Thematic analysis, using inductive coding | Method triangulation |
| Pawlowski et al. 2019b [20],  **Denmark** | Public health: PA promotion | To investigate girls’ perceptions of physical environmental factors influencing recess  physical activity in re-designed schoolyards and to compare the perceptions of girls from different age groups. | Process evaluation of quasi-experimental intervention,  Social constructionism | Schools: Recruited as part of larger project, schools selected from across Denmark.  Participants: Convenience sampling of children whose parents had consented at intervention schools. | Elementary & Lower Secondary (n=**5**),  urban & rural,  High & Low SES, Small & large student population & M^2^. | Students (n=**50**),  10-13 years,  100% female | Photo elicitation  Interviews, semi-structured, individual, and paired | Content analysis using pen profiles | Co-operative triangulation, critical friends |
| Pearce et al. 2011 [21],  **England** | Child development and play | To gather evidence of children’s perceptions of their playgrounds and play times. | Child-oriented research method:  Mosaic approach. | Schools: NR  Participants: Convenience sampling - all children at school invited to participate. | Elementary (n=**1**),  urban (London),  High SES, 12% minority ethnic groups, only classes up to Grade 4. | Students (n=**124**),  5-9 years,  51% female | Photo-elicitation, walking tour of playground, drawing task,  FG (subset of children) | Content analysis, using selective coding | Member checking, analytical audit*,* investigator triangulation |
| Powell et al. 2016 [22],  **England** | Public health: PA promotion | To assess the diversity of children's outdoor recess PA and explore children’s perceptions and experiences in relation to their playground environment. | Mixed methods, convergent parallel, Phenomenology, ideography, hermeneutics, SEM | Schools: Systematic and purposive sampling.  Participants: Random and stratified sampling from children whose parents had consented. | Elementary (n=**5**),  urban, West Midlands, Low SES. | Students (n=**80**),  7-10 years,  41% female | FG, semi-structured | Interpretive phenomenological analysis | Critical friends |
| Rasmussen et al. 2004 [23],  **Denmark** | Child development and play | To gain insight into the ways children experience institutionalized childhood and everyday life in the Danish welfare state | Sociology of childhood, Concept of place | Schools: Recruited as part of two projects within a larger program: ‘Childhood and Welfare Society’.  Participants: NR | Elementary (n=**NR**),  17 locations in Denmark (north, south, east, and west; city,  suburbs, provincial town & rural). | Students (n=**148**),  5-12 years | Photo elicitation (children’s photos of their favorite places),  Walking interviews | NR | NR |
| Ren et al. 2010 [24],  **USA ^ⴕ^** | Psychology | To work with children to assess and improve the institutional setting of recess. | Participatory action research design, Collaborative  community psychology | Schools: Invitation by Principal to help reform the playground.  Participants: Theoretical sampling, extreme and normative case sampling. | Elementary (n=**1**). | Students (n=**30**),  7-11 years | Observations  FG, semi-structured | Data coded inductively; themes developed | Data source triangulation, investigator triangulation, inter-rater reliability |
| Sharkey et al. 2014 [25],  **USA ^ⴕ^** | Education | To conduct a needs assessment and develop a customized training for yard supervisors at a public elementary school to help reduce student conflicts and promote a positive school climate. | Formative evaluation (Needs Assessment) | Schools: NR  Participants: NR | Elementary (n=**1**),  Central California,  Low SES. | Students (n=**NR**),  5-12 years,  43% Latino/a | FG, semi-structured | Data coded inductively; themes developed | Peer debrief, collaborative review of themes |
| Snow et al. 2019 [26],  **Australia** | Child development and play | To explore the perspectives of girls relating to the ideal school playground experience, and whether their perspectives are influenced by a loose-parts playground | Exploratory study design,  Affordance theory, Occupational justice | Schools: Recruited as part of a larger SPP project.  Participants: Purposive criterion sampling (gender, age, liked drawing and recess activities other than sport). | Elementary (n=**4**),  urban, Sydney, Med to High SES. | Students (n=**22**),  8-10 years,  100% female | Drawings,  Interviews, semi-structured,  Focus groups | Thematic analysis, using Braun & Clarke’s 6-step process | Investigator triangulation, inter-rater reliability,  collaborative review of themes |
| Stanley et al. 2012 [27],  **Australia** | Public health: PA promotion | To explore children’s perceptions of the factors influencing their engagement in physical activity during lunch break. | In-depth descriptive qualitative design,  SEM | Schools: Purposively selected from stratified list (school type, SES, location).  Participants: Maximum variation purposive sampling (age, gender, activity level, comfortable with FG method). | Elementary (n=**6**),  urban & rural, South Australia, Mix of school types, SES, geographic location. | Students (n=**54**),  10-13 years,  57% female,  20% Low SES, Active and not very active | FG, semi-structured,  Map-drawing | Content analysis, using a long table inductive thematic approach | Pilot FG, Investigator triangulation, negative case analysis, data source triangulation |
| Thomson et al. 2003 [28],  **England ^ⴕ^** | Child development and play | To explore the nature of contemporary primary school playground activities and whether these activities reflect the definition of play. | NR | Schools: NR  Participants: NR | Elementary (n=**4**), urban, suburban, and rural,  Variety of SES. | Students (n=NR) | Observation in playground,  Interviews, informal | NR | NR |
| Thomson et al. 2005 [29],  **England ^ⴕ^** | Human geography | To examine the spatial strategies used by playground supervisors, and present children’s territorialization of the playground and  responses to adults’ spatial control. | Ethnography,  Theory of human territoriality | Schools: Stratified random selection based on urban, suburban and rural location within a defined geographical area.  Participants: NR | Elementary (n=**3**), 1 each urban, suburban, and rural, Northern England & Midlands,  Low – Med SES. | Students (n=NR),  4-11 years | Ethnographic and  Systematic observation using RPI tool,  Field notes, Photographs,  Interviews, semi-structured and informal | Ethnographic analysis | Immersion in research context across three years |
| Thomson et al. 2007 [30],  **England ^ⴕ^** | Education | To elucidate children’s experience of the school playground environment and explore how adults limit children's interaction with the environment. | Ethnography,  Free choice learning | Schools: Stratified random selection based on urban, suburban and rural location within a defined geographical area.  Participants: NR | Elementary (n=**3**), 1 each urban, suburban, and rural, Northern England & Midlands,  Low – Med SES. | Students (n=NR),  4-11 years | Ethnographic and  Systematic observation using RPI tool,  Field notes,  Photographs,  Interviews, semi-structured and informal | Ethnographic analysis | Immersion in research context across three years |
| Willenberg et al. 2010 [31],  **Australia** | Public health: PA promotion | To increase understanding of how children respond to their school playground environments. | Mixed methods,  Sociology of childhood | Schools: Randomized - part of a cRCT ‘fun ‘n healthy in Moreland!’ project.  Participants: Purposive sampling based on age. | Elementary (n=**12**),  urban, government, independent, religious, and special development schools, Low SES. | Students (n=**91**),  8-11 years | FG, involving concept mapping, group discussion, drawing, photo ordering technique. | Data coded inductively; themes developed | Investigator triangulation, collaborative review of themes. |

Legend: cRCT = cluster Randomised Controlled Trial; EnRG = Environmental Research framework for weight Gain prevention; FG = Focus Group; GRF-OT = Great Recess Framework–Observational Tool; HEPCOM= Promoting Healthy Eating and Physical Activity in local Communities; Med = Medium; N/A = Not Applicable; NASN = National Association of School Nurses; NR = Not Reported; NOW Play = Northern Oral language and Writing through Play; NPLS = Nature Play and Learning Spaces; NW = North-western; PA = Physical Activity; RCT = Randomised Controlled Trial; RPI = Record of Perceived Intervention; SCT = Social Cognitive Theory; SDT = Social Determination Theory; SEM = Social Ecological Model; SES = Socio-Economic Status; SPP = Sydney Playground Project; STEAM = Spatial Temporal Environmental and Activity Monitoring.

**^ⴕ^** Studies involved both adult and child participants, however, only child population and methods described.

# References

1. Button, B.L., S. Tillmann, and J. Gilliland, *Exploring children's perceptions of barriers and facilitators to physical activity in rural Northwestern Ontario, Canada.* Rural and Remote Health, 2020. **20**(3): p. 5791-5791.

2. Caro, H.E.E., et al., *Dutch Primary Schoolchildren's Perspectives of Activity-Friendly School Playgrounds: A Participatory Study.* International journal of environmental research and public health, 2016. **13**(6): p. 526.

3. Christensen, P. and M.R. Mikkelsen, *Jumping off and being careful: Children's strategies of risk management in everyday life.* Sociology of Health and Illness, 2008. **30**(1): p. 112-130.

4. Clements, R., M. Messanga, and A.-M. Millbank, *Traditional Children's Games in Tanzania.* Children, Youth & Environments, 2008. **18**(2): p. 206-218.

5. Eskola, S., et al., *Children's perceptions of factors related to physical activity in schools.* Educational Research, 2018. **60**(4): p. 410-426.

6. Gyllencreutz, L., et al., *Injury risks during outdoor play among Swedish schoolchildren: teachers’ perceptions and injury preventive practices.* Education 3-13, 2020. **48**(1): p. 1-11.

7. Harvey, J., et al., *Exploring the perspectives of 10-, 11-, and 12-year-old primary school students on physical activity engagement—“'Cause you can't just be sitting at a desk all the time!”.* Child: Care, Health and Development, 2018. **44**(3): p. 433-442.

8. Hemming, P., *Renegotiating the Primary School: Children's Emotional Geographies of Sport, Exercise and Active Play.* Children's Geographies, 2007. **5**(4): p. 353-371.

9. Hyndman, B., et al., *Moving Physical Activity Beyond the School Classroom: A Social-ecological Insight for Teachers of the facilitators and barriers to students' non-curricular physical activity.* Australian Journal of Teacher Education, 2012. **37**(2): p. 1.

10. Hyndman, B.P. and A. Telford, *Should educators be ‘wrapping school playgrounds in cotton wool’to encourage physical activity? Exploring primary and secondary students’ voices from the school playground.* Australian Journal of Teacher Education, 2015. **40**(6): p. 60-84.

11. Jarvis, P., *Dangerous Activities within an Invisible Playground: A Study of Emergent Male Football Play and Teachers' Perspectives of Outdoor Free Play in the Early Years of Primary School.* International Journal of Early Years Education, 2007. **15**(3): p. 245-259.

12. Martínez-Andrés, M., et al., *“Football is a boys’ game”: children’s perceptions about barriers for physical activity during recess time.* International Journal of Qualitative Studies on Health and Well-being, 2017. **12**(1): p. 1379338.

13. Massey, W.V., et al., *Observations from the playground: Common problems and potential solutions for school-based recess.* Health Education Journal, 2020.

14. McNamara, L., *What's Getting in the Way of Play? An Analysis of the Contextual Factors that Hinder Recess in Elementary Schools.* Canadian Journal of Action Research, 2013. **14**(2): p. 3-21.

15. McWhannell, N., C. Triggs, and S. Moss, *Perceptions and measurement of playtime physical activity in English primary school children: The influence of socioeconomic status.* European Physical Education Review, 2019. **25**(2): p. 438-455.

16. Ndhlovu, S. and V. Varea, *Primary School Playgrounds as Spaces of Inclusion/Exclusion in New South Wales, Australia.* Education 3-13, 2018. **46**(5): p. 494-505.

17. Norðdahl, K. and J. Einarsdóttir, *Children's Views and Preferences Regarding Their Outdoor Environment.* Journal of Adventure Education and Outdoor Learning, 2015. **15**(2): p. 152-167.

18. Parrish, A.M., et al., *Using interviews and peer pairs to better understand how school environments affect young children's playground physical activity levels: a qualitative study.* Health Education Research, 2012. **27**(2): p. 269-280.

19. Pawlowski, C.S., et al., *Changing recess geographies: children’s perceptions of a schoolyard renovation project promoting physical activity.* Children's Geographies, 2019. **17**(6): p. 664-675.

20. Pawlowski, S.C., et al., *Designing Activating Schoolyards: Seen from the Girls’ Viewpoint.* International Journal of Environmental Research and Public Health, 2019. **16**(19).

21. Pearce, G. and R.P. Bailey, *Football pitches and Barbie dolls: young children’s perceptions of their school playground.* Early Child Development and Care, 2011. **181**(10): p. 1361-1379.

22. Powell, E., L.A. Woodfield, and A.A.M. Nevill, *Children’s physical activity levels during primary school break times: A quantitative and qualitative research design.* European Physical Education Review, 2016. **22**(1): p. 82-98.

23. Rasmussen, K., *Places for Children - Children's Places.* Childhood: A Global Journal of Child Research, 2004. **11**(2): p. 155-173.

24. Ren, J.Y. and R.D. Langhout, *A recess evaluation with the players: taking steps toward participatory action research.* American journal of community psychology, 2010. **46**(1-2): p. 124-138.

25. Sharkey, J.D., et al., *Effective yard supervision: From needs assessment to customized training.* Contemporary School Psychology, 2014. **18**(2): p. 103-116.

26. Snow, D., et al., *Girls’ perspectives on the ideal school playground experience: an exploratory study of four Australian primary schools.* Children's Geographies, 2019. **17**(2): p. 148-161.

27. Stanley, R.M., K. Boshoff, and J. Dollman, *Voices in the playground: A qualitative exploration of the barriers and facilitators of lunchtime play.* Journal of Science and Medicine in Sport, 2012. **15**(1): p. 44-51.

28. Thomson, S., *A well-equipped hamster cage: The rationalisation of primary school playtime.* Education 3-13, 2003. **31**(2): p. 54-59.

29. Thomson, S., *‘Territorialising’ the primary school playground: deconstructing the geography of playtime.* Children's Geographies, 2005. **3**(1): p. 63-78.

30. Thomson, S., *Do’s and don’ts: children’s experiences of the primary school playground.* Environmental Education Research, 2007. **13**(4): p. 487-500.

31. Willenberg, L.J., et al., *Increasing school playground physical activity: A mixed methods study combining environmental measures and children's perspectives.* Journal of Science and Medicine in Sport, 2010. **13**(2): p. 210-216.
