# Supplementary material for: “All the fun stuff, the teachers say, ‘that’s dangerous!’” Hearing from children on safety and risk in active play in schools: a systematic review
Source: Int J Behav Nutr Phys Act. 2022 Jun 25;19:72. doi: 10.1186/s12966-022-01305-0 (PMC9233824; doi:10.1186/s12966-022-01305-0)
Supplement: Supplementary file 5 — Additionalfile 5. Quality appraisal of included studies. Table showing appraisal results for all studies using the CASP checklist. [file 12966_2022_1305_MOESM5_ESM.docx]

# Additional file 5: Quality Appraisal of included studies

|  | **SCREENING QUESTIONS** | | **APPRAISAL QUESTIONS** | | | | | | | |
| --- | --- | --- | --- | --- | --- | --- | --- | --- | --- | --- |
| **CASP CHECKLIST** | **1. Aims of study** | **2. Appropriateness of qualitative methodology** | **3. Research design** | **4. Recruitment strategy** | **5. Data collection** | **6. Reflexivity-related issues** | **7. Ethical issues** | **8. Rigor of data analysis** | **9. Reporting of study findings** | **10. Value of study findings** |
| Button et al. 2020 | 1 | 1 | 1 | 1 | 1 | 1 | 1 | 1 | 1 | 1 |
| Caro et al. 2016 | 1 | 1 | 1 | 1 | 1 | 1 | 1 | 1 | 1 | 1 |
| Christiansen et al. 2008 | 1 | 1 | 1 | 0.5 | 1 | 0 | 1 | 0.5 | 0.5 | 0.5 |
| Clements et al. 2008 | 1 | 1 | 0 | 0 | 0 | 0 | 0 | 0 | 0.5 | 0.5 |
| Eskola et al. 2018 | 1 | 1 | 1 | 1 | 1 | 0 | 1 | 1 | 1 | 1 |
| Gyllencreutz et al. 2020 | 1 | 1 | 1 | 0.5 | 1 | 0 | 1 | 1 | 0.5 | 1 |
| Harvey et al. 2018 | 1 | 1 | 1 | 1 | 1 | 0 | 1 | 1 | 1 | 1 |
| Hemming et al. 2007 | 1 | 1 | 1 | 0.5 | 1 | 1 | 1 | 0 | 1 | 1 |
| Hyndman et al. 2012 | 1 | 1 | 1 | 1 | 1 | 0 | 0.5 | 1 | 1 | 1 |
| Hyndman et al. 2015 | 1 | 1 | 1 | 1 | 1 | 0 | 1 | 1 | 1 | 1 |
| Jarvis et al. 2007 | 0.5 | 1 | 1 | 0 | 1 | 1 | 0 | 1 | 1 | 1 |
| Martinez et al. 2017 | 1 | 1 | 1 | 1 | 1 | 0 | 1 | 1 | 1 | 1 |
| Massey et al. 2020 | 1 | 1 | 1 | 0 | 1 | 1 | 0.5 | 1 | 1 | 1 |
| McNamara et al. 2013 | 1 | 1 | 1 | 1 | 1 | 0.5 | 0 | 1 | 1 | 1 |
| McWhannel et al. 2019 | 1 | 1 | 1 | 1 | 1 | 1 | 1 | 1 | 1 | 1 |
| Ndhlovu et al. 2018 | 1 | 1 | 1 | 1 | 1 | 0 | 1 | 1 | 1 | 1 |
| Norodahl et al. 2015 | 1 | 1 | 1 | 1 | 1 | 0 | 1 | 1 | 1 | 1 |
| Parrish et al. 2012 | 1 | 1 | 1 | 1 | 1 | 0 | 1 | 0.5 | 1 | 1 |
| Pawlowski et al. 2019 | 1 | 1 | 1 | 1 | 1 | 0 | 1 | 1 | 1 | 1 |
| Pawlowski et al. 2019 | 1 | 1 | 1 | 1 | 1 | 0 | 1 | 1 | 1 | 1 |
| Pearce et al. 2011 | 1 | 1 | 1 | 1 | 1 | 1 | 1 | 1 | 1 | 1 |
| Powell et al. 2016 | 1 | 1 | 1 | 1 | 1 | 0 | 1 | 1 | 1 | 1 |
| Rasmussen et al. 2004 | 0.5 | 1 | 0 | 0 | 0 | 0 | 0 | 0 | 0.5 | 0.5 |
| Ren et al. 2010 | 1 | 1 | 1 | 1 | 1 | 0 | 1 | 1 | 1 | 1 |
| Sharkey et al. 2014 | 1 | 1 | 1 | 0 | 1 | 0 | 0.5 | 1 | 1 | 1 |
| Snow et al. 2019 | 1 | 1 | 1 | 1 | 1 | 0 | 1 | 1 | 1 | 1 |
| Stanley et al. 2012 | 1 | 1 | 1 | 1 | 1 | 1 | 1 | 1 | 1 | 1 |
| Thomson et al. 2003 | 0.5 | 0 | 0.5 | 0 | 0 | 0 | 0 | 0 | 0.5 | 0.5 |
| Thomson et al. 2005 | 1 | 1 | 0.5 | 1 | 1 | 0 | 1 | 1 | 0.5 | 1 |
| Thomson et al. 2007 | 1 | 1 | 1 | 1 | 1 | 1 | 1 | 0.5 | 1 | 1 |
| Willenberg et al. 2010 | 1 | 1 | 1 | 1 | 1 | 0 | 0.5 | 0.5 | 1 | 1 |

Legend: Totally met = 1; Somewhat met = 0.5; Not met = 0; Can’t tell = 0

Consistent with recently published approaches [1], the CASP Checklist was modified to include ‘Somewhat’, where an item was partially met, in addition to the ‘Yes’ (totally met) ‘No’ (not met) and ‘Can’t tell’ (not enough information to make a judgement) options.

**References**

1. Long, H.A., D.P. French, and J.M. Brooks, *Optimising the value of the critical appraisal skills programme (CASP) tool for quality appraisal in qualitative evidence synthesis.* Research Methods in Medicine & Health Sciences, 2020. **1**(1): p. 31-42.
